# Recurrent Pulmonary Tuberculosis in China, 2005 to 2021

**DOI:** 10.1001/jamanetworkopen.2024.27266

**Published:** 2024-08-12

**Authors:** Tao Li, Bo Zhang, Xin Du, Shaojun Pei, Zhongwei Jia, Yanlin Zhao

**Affiliations:** 1 Department of Epidemiology and Biostatistics, School of Public Health, Peking University, Beijing, China; 2 National Center for Tuberculosis Control and Prevention, Chinese Center for Disease Control and Prevention, Beijing, China; 3 National Key Laboratory of Intelligent Tracking and Forecasting for Infectious Diseases, Chinese Center for Disease Control and Prevention, Beijing, China; 4 Center for Intelligent Public Health, Institute for Artificial Intelligence, Peking University, Beijing, China; 5 School of Environmental Science and Engineering, Hainan University, Haikou, China; 6 Center for Drug Abuse Control and Prevention, National Institute of Health Data Science, Peking University, Beijing, China; 7 Department of Global Health, School of Public Health, Peking University, Beijing, China

## Abstract

**Importance:**

Despite posing a significant challenge to global tuberculosis (TB) elimination efforts, recurrent TB remains understudied due to the challenges of long-term observation.

**Objective:**

To investigate the burden of recurrent TB using data from patients with pulmonary TB (PTB) in China.

**Design, Setting, and Participants:**

This retrospective cohort study included all bacteriologically confirmed or clinically diagnosed PTB cases reported to the Tuberculosis Information Management System with completed or successful treatment outcomes from January 1, 2005, to December 31, 2021. Data were analyzed from July 15, 2022, to October 28, 2023.

**Exposures:**

Newly diagnosed PTB was classified into primary, hematogenous disseminated, or secondary PTB.

**Main Outcomes and Measures:**

The primary outcome was the annual recurrence rate, stratified by disease classification, over the 17-year observation period. The recurrence rate for year *n* was calculated by dividing the number of patients with recurrent TB in year *n* by observed person-years in year *n*. The secondary outcome was the annual proportion of recurrent TB among reported cases and associated risk factors.

**Results:**

Of 13 833 249 patients with TB reported to the Tuberculosis Information Management System, 10 482 271 with PTB met the inclusion criteria. Of these, 68.9% were male, 22.3% were 65 years or older, 89.6% were of Han ethnicity, and 68.4% were agricultural workers. A total of 413 936 patients experienced a recurrent TB episode after successful treatment, resulting in an overall recurrence rate of 0.47 (95% CI, 0.47-0.48) per 100 person-years. The recurrence rate for patients with primary PTB was 0.24 (95% CI, 0.22-0.26) per 100 person-years; for hematogenous disseminated PTB, 0.37 (95% CI, 0.36-0.38) per 100 person-years; and for secondary PTB, 0.48 (95% CI, 0.47-0.48) per 100 person-years. The cumulative proportion of recurrences within the first 2 years accounted for 48.9% of all recurrent cases. The proportion of recurrent cases among notified incident cases increased 1.9-fold from 4.7% in 2015 to 8.8% in 2021. Among other factors, ages 45 to 64 years (adjusted hazard ratio, 1.77 [95% CI, 1.65-1.89]) and having completed treatment (adjusted hazard ratio, 1.16 [95% CI, 1.14-1.18]) were identified as associated with recurrence.

**Conclusions and Relevance:**

In this retrospective cohort study, the PTB recurrence rate was substantially higher than the incidence, and the proportion of recurrent cases increased. Almost half of the recurrence occurred within the first 2 years, suggesting that routine posttreatment follow-up may represent an important strategy for accelerating TB elimination.

## Key Points

**Question:**

What is the epidemiological burden of recurrent tuberculosis (TB) in China?

**Findings:**

In this cohort study of more than 10 million patients with pulmonary TB, the overall recurrence rate was 0.47 per 100 person-years, and half of the recurrent events occurred within the first 2 years after treatment completion. The proportion of recurrent cases among incident cases reported to the notification system increased 2-fold from 2015 to 2021, and the mean recurrence risk was 8 times higher than the estimated incidence.

**Meaning:**

These findings suggest that implementation of routine posttreatment follow-up may be a viable strategy for accelerating TB elimination.

## Teaser

This cohort study uses a national reporting system to calculate the rate of recurrence among patients with confirmed incident cases of pulmonary tuberculosis in China.

## Introduction

Recurrent tuberculosis (TB), or relapse TB as defined by the World Health Organization (WHO),^1^ occurs in patients who have previously been treated for TB, were declared cured or completed treatment at the end of their most recent course, and are subsequently diagnosed with a recurrent episode of TB (either a true relapse or a new episode caused by reinfection). Molecular biology techniques, such as whole genome sequencing,^2^ can be used to differentiate between relapse (reactivation) and reinfection; however, the results are not always definitive. Recurrence poses a substantial challenge to the implementation and achievement of the global End TB strategy.^3^ Lambert et al^4^ reported that approximately 5% of patients with drug-susceptible TB may develop another episode of TB after 6 months of first-line therapy. In 2021, over 350 000 cases of recurrent TB were reported globally, accounting for 5.5% of all 6.4 million reported incident cases.^5^

A systematic review of 145 studies with a mean posttreatment follow-up duration of 2.3 years^6^ reported a pooled recurrence rate of 2.26 (95% CI, 1.87-2.73) per 100 person-years at risk and found that this rate was higher in countries with high TB incidence compared with low TB incidence (4.10 [95% CI, 2.67-6.28] vs 1.47 [95% CI, 0.87-2.46] per 100 person-years). Most studies on long-term TB recurrence have been conducted in low-incidence countries (eg, Finland,^7^ the Netherlands,^8^ and the US^9^) and have not clearly described the recurrence risk pattern over time. A population-level cohort study in South Africa (2003-2016)^10^ found high TB recurrence rates (16.4 [95% CI, 16.2-16.6] per 1000 person-years) that increased per subsequent episode. China, which has a high TB burden with the third-highest incidence in the world, is also facing a significant recurrence challenge. A study from Xinjiang province^11^ found that nearly 20% of patients with newly diagnosed pulmonary TB (PTB) may experience a recurrent episode within 5 years of successful treatment. Another study from Jiangsu, a low-incidence province,^12^ found a comparatively lower recurrence rate than in Xinjiang, but this rate was still 25 times higher than in the general population. A national survey in China^13^ found that 25.6% of patients with a history of TB treatment may have multidrug-resistant TB (MDR-TB).

In 2005, China implemented the nationwide, web-based Tuberculosis Information Management System (TBIMS) based on the national Infectious Diseases Reporting System launched after SARS.^14^ With its high horizontal and vertical coverage, the TBIMS provides continuous and reliable notification data at both national and subnational levels. It is the largest TB notification system in the world, covering all TB control facilities in China’s 31 mainland provinces, over 300 prefectures, and 3000 counties.^14^ The TBIMS is considered a comprehensive TB monitoring system and has been recommended by the WHO as an example for global TB information surveillance systems.^15^ In China, all individuals with bacteriological confirmation or a clinical diagnosis of TB are required to be reported to the TBIMS, and a national survey found that 91.7% of patients diagnosed with PTB in 2015 were reported to the TBIMS.^16^ The TBIMS collects data on patient demographic characteristics, diagnosis, treatment, and full-course management information from designated national TB program health facilities at all levels. Logic validation rules are applied during data collection, and outliers are corrected to meet system requirements. Missing values are processed using the dummy variables.

 Although the Han ethnic group accounts for the majority of the general population of China, other ethnic groups are still an important component, and previous studies have reported differences in TB burden between Han individuals and other ethnic minority individuals.^46^ Clarifying this aspect of TB incidence will help strengthen efforts in subsequent work to address inequalities among ethnic groups, and the TBIMS provides a unique opportunity to conduct long-term observation and study TB recurrence in China.

## Methods

This cohort study was reviewed and approved by the Ethical Review Committee of the Chinese Center for Disease Control and Prevention. As this study involved routinely collected secondary data, the review board waived the requirement for informed consent. The study followed the Strengthening the Reporting of Observational Studies in Epidemiology (STROBE) reporting guideline.

### Study Design and Procedures

We used data extracted from the TBIMS in April 2022 to assess TB recurrence rates over time among patients with newly diagnosed PTB, stratified by TB classifications, across mainland China. Data were collected between January 1, 2005, and December 31, 2021. The inclusion criteria were as follows: (1) pulmonary lesions categorized as primary, hematogenous disseminated, or secondary PTB; (2) newly diagnosed TB for the first treatment episode; and (3) classified as being cured or completing treatment as outcomes for their first treatment episode. The exclusion criteria were as follows: (1) missing a treatment end date; (2) no pulmonary lesions (categorized as any kind of extrapulmonary alone); (3) classified as re-treated for their first treatment episode; and (4) any other unsuccessful treatment outcomes for the first treatment episode.

### Outcomes and Definitions

The primary outcome was the annual recurrence rate in patients with newly diagnosed PTB over the 17-year observation period, stratified by disease classification. The secondary outcome was the proportion of recurrent TB among reported TB cases and associated risk factors, as well as the proportion of incident cases that were recurrent, disaggregated by year. The date of TB recurrence was defined as the confirmation date. Patient delay was defined as the interval between symptom onset and first medical visit. Diagnosis delay was defined as the interval between first medical visit and diagnosis confirmation.

The diagnosis and classification of PTB were based on the national criteria outlined by the National Health and Family Planning Commission.^17^ Pulmonary TB included confirmed cases, clinically diagnosed cases, and presumptive cases based on etiological test results in combination with epidemiological history, clinical symptoms, chest imaging, and other relevant auxiliary tests. However, only confirmed and clinically diagnosed cases are reported to the TBIMS. Pulmonary TB was classified as primary, hematogenous disseminated, or secondary (including tracheobronchial TB). Recurrent TB was defined according to the WHO definition.^1^ Patients with a prior successful treatment history (declared as cured or treatment completed at the end of treatment course) who subsequently had a clinical diagnosis or laboratory confirmation of a new TB episode were categorized as having recurrent TB. The definitions of *cured* and *treatment completed* were consistent with WHO surveillance framework^1^ and national TB control guidelines.^18^

Treatment regimens were categorized into 5 groups^18^: standardized treatment (2 months of isoniazid, rifampicin, pyrazinamide, and ethambutol [2HRZE], followed by 4 months of isoniazid and rifampicin [4HR]), intensive treatment (2HRZE, followed by 7-10 months of isoniazid, rifampicin, and ethambutol [7-10HRE]), complications or severe treatment (2HRZE/10HRE), personalized treatment regimen, and MDR-TB treatment. Standardized treatment consisted of 6 months of daily first-line anti-TB drug treatment. Intensive treatment and treatment for complications and severe disease were both based on standardized treatment. Personalized regimens were based on the latest treatment guidelines of the WHO^19^ and were adjusted for individual patient characteristics. The drug-resistant regimen was based on the WHO-recommended, long-term MDR-TB treatment regimen.^19^

### Probabilistic Matching Algorithm

The national identifier number was not a mandatory data element in the TBIMS until 2016. Therefore, we developed a probabilistic algorithm^20^ to identify individual patients based on their unique identifier number or a combination of distinguishing variables. Records without an identifier number were matched using name plus date of birth plus domicile code by calculating their similarity scores. Our algorithm effectively avoided overmatching (ie, linkage of records that are not true matches) and undermatching (ie, failure to link true matches). We found a sensitivity of 91.0% (ie, 9.0% undermatching) and specificity of 90.0% (ie, 10.0% overmatching). The detailed process of records matching is described in the eAppendix in Supplement 1.

### Statistical Analysis

Data were analyzed from July 15, 2022, to October 28, 2023. The recurrence rate of year *n* (ie, years since enrollment) was calculated by dividing the number of patients with recurrence in year *n* by the observed person-years in year *n*. Kaplan-Meier analysis was used to calculate the survival probabilities for successfully treated patients who had not experienced a recurrence, by year. Cox proportional hazards regression models were used to compare recurrence rates among subgroups. Patients without recurrence were right censored on December 31, 2021. The Schoenfeld residuals for each covariate were assessed using the Survminer package (R, version 4.2.3 [R Project for Statistical Computing]) to confirm that the proportional hazards assumption was met (eFigure 5 in Supplement 1). To determine whether the recurrence rate differed according to patient characteristics, such as regimens, we performed stratified, multivariable Cox proportional hazards regression analyses to estimate hazard ratios (HRs) reported with 95% CIs.

A 2-sided *P* ≤ .05 was regarded as statistically significant. Data were double checked in both a relational database designed in SQL Server 2008 (Microsoft Corporation) and in SPSS, version 17.0 (IBM Corp). Statistical analyses were performed with R software, version 4.2.3. The probabilistic matching algorithm was developed by Java, version 1.8.0 (Oracle).

## Results

As of December 31, 2021, the TBIMS included records for 13 833 249 patients with TB. After excluding 648 459 patients with extrapulmonary TB, 888 449 were classified as re-treated in their first record, 1 249 825 did not have a treatment end date, and 564 245 had unsuccessful treatment outcomes, leaving 10 482 271 patients with newly diagnosed PTB who were included in the study cohort (eFigure 1 in Supplement 1).

Among the 10 482 271 patients with PTB included in the study, 7 227 196 (68.9%) were male and 3 255 075 (31.1%) were female, 2 332 596 (22.3%) were 65 years or older, 9 389 889 (89.6%) were of Han ethnicity, 7 165 802 (68.4%) were agricultural workers, 886 681 (8.5%) were migrants, 4 631 413 (44.2%) had bacteriologically confirmed TB, 788 084 (7.5%) had undergone drug sensitivity tests, and 783 467 (7.5%) had drug-susceptible TB. In addition, 9 560 919 patients (91.2%) received standardized treatment (2HRZE/4HR), 7 613 312 (72.6%) received treatment at disease control institutions, 5 767 220 (55.0%) received full-course supervision, 4 341 306 (41.4%) were enrolled from a direct visit, 4 505 800 (43.0%) were enrolled from referral, 5 974 364 (57.0%) completed treatment, 5 109 881 (48.7%) had a patient delay of more than 4 weeks, and 2 540 229 (24.2%) had a diagnostic delay of more than 1 week (Table 1).

**Table 1.** Recurrence Rates of Patients With Newly Diagnosed PTB in China, 2005 to 2021

Characteristic	Total			Primary PTB			Hematogenous disseminated PTB			Secondary PTB		
	Total No. (%)	Recurrence, No. (%)	Rate/100 PY	No. (%)	Recurrence, No. (%)	Rate/100 PY	No. (%)	Recurrence, No. (%)	Rate/100 PY	No. (%)	Recurrence, No. (%)	Rate/100 PY
Total	10 482 271 (100)	413 936 (100)	0.47	24 557 (100)	584 (100)	0.24	114 080 (100)	3834 (100)	0.37	10 343 634 (100)	409 518 (100)	0.48
Sex												
Male	7 227 196 (68.9)	303 486 (73.3)	0.50	14 708 (59.9)	349 (59.8)	0.23	74 430 (65.2)	2736 (71.4)	0.40	7 138 058 (69.0)	300 401 (73.4)	0.50
Female	3 255 075 (31.1)	110 450 (26.7)	0.41	9849 (40.1)	235 (40.2)	0.25	39 650 (34.8)	1098 (28.6)	0.30	3 205 576 (31.0)	109 117 (26.6)	0.41
Age, y												
0-4	6203 (0.1)	26 (0.01)	0.04	2088 (8.5)	10 (1.7)	0.04	371 (0.3)	5 (0.1)	0.14	3744 (0.04)	11 (0.003)	0.03
5-14	42 229 (0.4)	908 (0.2)	0.24	8702 (35.4)	154 (26.4)	0.15	1716 (1.5)	39 (1.0)	0.25	31 811 (0.3)	715 (0.2)	0.27
15-24	1 474 866 (14.1)	54 337 (13.1)	0.42	5874 (23.9)	176 (30.1)	0.32	20 170 (17.7)	744 (19.4)	0.39	1 448 822 (14.0)	53 417 (13.0)	0.42
25-44	3 154 225 (30.1)	107 573 (26.0)	0.39	3912 (15.9)	81 (13.9)	0.24	35 798 (31.4)	1165 (30.4)	0.34	3 114 515 (30.1)	106 327 (26.0)	0.39
45-64	3 472 152 (33.1)	159 429 (38.5)	0.57	2709 (11.0)	104 (17.8)	0.50	34 336 (30.1)	1288 (33.6)	0.42	3 435 107 (33.2)	158 037 (38.6)	0.57
65-84	2 229 954 (21.3)	89 450 (21.6)	0.51	1224 (5.0)	59 (10.1)	0.65	20 829 (18.3)	579 (15.1)	0.33	2 207 901 (21.3)	88 812 (21.7)	0.51
≥85	102 642 (1.0)	2213 (0.5)	0.32	48 (0.2)	0	0	860 (0.8)	14 (0.4)	0.24	101 734 (1.0)	2199 (0.5)	0.32
Ethnicity												
Han	9 389 889 (89.6)	350 120 (84.6)	0.43	18 449 (75.1)	411 (70.4)	0.19	89 254 (78.2)	2899 (75.6)	0.33	9 282 186 (89.7)	346 810 (84.7)	0.44
Ethnic minority group^a^	1 088 306 (10.4)	63 719 (15.4)	0.93	6102 (24.8)	173 (29.6)	0.51	24 799 (21.7)	933 (24.3)	0.56	1 057 405 (10.2)	62 613 (15.3)	0.94
Unknown	4076 (0.01)	97 (0.02)	0.41	6 (0.02)	0	0	27 (0.02)	2 (0.1)	1.14	4043 (0.04)	95 (0.02)	0.40
Occupation												
Preschooler	6235 (0.1)	78 (0.02)	0.15	2211 (9.0)	19 (3.3)	0.08	502 (0.4)	6 (0.2)	0.16	3522 (0.0)	53 (0.01)	0.22
Student	553 900 (5.3)	17 008 (4.1)	0.37	11 643 (47.4)	247 (42.3)	0.19	7851 (6.9)	274 (7.1)	0.39	534 406 (5.2)	16 487 (4.0)	0.38
Industrial worker	525 157 (5.0)	19 197 (4.6)	0.40	452 (1.8)	14 (2.4)	0.31	3568 (3.1)	115 (3.0)	0.32	521 137 (5.0)	19 068 (4.7)	0.40
Agricultural worker	7 165 802 (68.4)	293 734 (71.0)	0.48	8015 (32.6)	231 (39.6)	0.33	85 924 (75.3)	2952 (77.0)	0.37	7 071 863 (68.4)	290 551 (70.9)	0.49
Commercial worker	172 420 (1.6)	5634 (1.4)	0.42	156 (0.6)	4 (0.7)	0.33	1152 (1.0)	42 (1.1)	0.41	171 112 (1.7)	5588 (1.4)	0.42
Health care worker	37 852 (0.4)	834 (0.2)	0.30	69 (0.3)	2 (0.3)	0.36	234 (0.2)	6 (0.2)	0.30	37 549 (0.4)	826 (0.2)	0.30
Retired	355 685 (3.4)	13 200 (3.2)	0.50	313 (1.3)	21 (3.6)	0.85	2490 (2.2)	72 (1.9)	0.36	352 882 (3.4)	13 107 (3.2)	0.50
Office worker	204 957 (2.0)	6010 (1.5)	0.36	262 (1.1)	4 (0.7)	0.17	1313 (1.2)	39 (1.0)	0.34	203 382 (2.0)	5967 (1.5)	0.36
Domestic duties or unemployed	958 417 (9.1)	39 685 (9.6)	0.59	639 (2.6)	21 (3.6)	0.47	6874 (6.0)	201 (5.2)	0.36	950 904 (9.2)	39 463 (9.6)	0.59
Other	501 846 (4.8)	18 556 (4.5)	0.40	797 (3.2)	21 (3.6)	0.31	4172 (3.7)	127 (3.3)	0.32	496 877 (4.8)	18 408 (4.5)	0.40
Migrant												
Yes	886 681 (8.5)	39 070 (9.4)	0.56	1072 (4.4)	31 (5.3)	0.34	8337 (7.3)	357 (9.3)	0.50	877 272 (8.5)	38 682 (9.4)	0.56
No	9 595 590 (91.5)	374 866 (90.6)	0.47	23 485 (95.6)	553 (94.7)	0.23	105 743 (92.7)	3477 (90.7)	0.36	9 466 362 (91.5)	370 836 (90.6)	0.47
Bacteriological test result												
Positive	4 631 413 (44.2)	207 474 (50.1)	0.50	3205 (13.1)	124 (21.2)	0.43	35 409 (31.0)	1343 (35.0)	0.40	4 592 799 (44.4)	206 007 (50.3)	0.50
Negative	5 798 241 (55.3)	204 973 (49.5)	0.45	15 767 (64.2)	369 (63.2)	0.24	77 077 (67.6)	2450 (63.9)	0.35	5 705 397 (55.2)	202 154 (49.4)	0.45
Unknown	52 617 (0.5)	1489 (0.4)	0.46	5585 (22.7)	91 (15.6)	0.15	1594 (1.4)	41 (1.1)	0.47	45 438 (0.4)	1357 (0.3)	0.53
Rifampin resistance												
Resistant	4617 (0.04)	422 (0.1)	2.93	4 (0.02)	1 (0.2)	29.41	15 (0.01)	2 (0.1)	5.92	4598 (0.04)	419 (0.1)	2.91
Sensitive	783 467 (7.5)	33 765 (8.2)	1.64	389 (1.6)	15 (2.6)	3.14	4799 (4.2)	127 (3.3)	1.07	778 279 (7.5)	33 623 (8.2)	1.64
Unknown	9 694 187 (92.5)	379 749 (91.7)	0.45	24 164 (98.4)	568 (97.3)	0.23	109 266 (95.8)	3705 (96.6)	0.36	9 560 757 (92.4)	375 476 (91.7)	0.45
Therapeutic regimen												
2HRZE/4HR	9 560 919 (91.2)	383 684 (92.7)	0.47	16 956 (69.0)	462 (79.1)	0.29	100 652 (88.2)	3536 (92.2)	0.37	9 443 311 (91.3)	379 686 (92.7)	0.48
2HRZE/7-10HRE	57 431 (0.5)	2078 (0.5)	0.51	297 (1.2)	7 (1.2)	0.53	1106 (1.0)	29 (0.8)	0.39	56 028 (0.5)	2042 (0.5)	0.51
2HRZE/10HRE	118 476 (1.1)	2570 (0.6)	0.83	139 (0.6)	2 (0.3)	0.68	2971 (2.6)	45 (1.2)	0.67	115 366 (1.1)	2523 (0.6)	0.84
Personalized	724 193 (6.9)	25 099 (6.1)	0.43	7136 (29.1)	113 (19.3)	0.14	9249 (8.1)	222 (5.8)	0.34	707 808 (6.8)	24 764 (6.0)	0.44
MDR	21 252 (0.2)	505 (0.1)	1.01	29 (0.1)	0	0	102 (0.1)	2 (0.1)	0.55	21 121 (0.2)	503 (0.1)	1.02
Treatment management institution												
Infectious disease hospital	710 690 (6.8)	25 382 (6.1)	0.57	932 (3.8)	12 (2.1)	0.17	5575 (4.9)	127 (3.3)	0.34	704 183 (6.8)	25 243 (6.2)	0.58
Primary health institution	57 575 (0.5)	2314 (0.6)	0.52	170 (0.7)	4 (0.7)	0.28	440 (0.4)	18 (0.5)	0.47	56 965 (0.6)	2292 (0.6)	0.52
Disease control institution	7 613 312 (72.6)	304 505 (73.6)	0.42	19 377 (78.9)	450 (77.1)	0.20	87 580 (76.8)	3105 (81.0)	0.34	7 506 355 (72.6)	300 950 (73.5)	0.42
General hospital	2 100 694 (20.0)	81 735 (19.7)	0.82	4078 (16.6)	118 (20.2)	0.87	20 485 (18.0)	584 (15.2)	0.62	2 076 131 (20.1)	81 033 (19.8)	0.82
Supervision pattern												
Full-course supervision^b^	5 767 220 (55.0)	256 776 (62.0)	0.51	5294 (21.6)	159 (27.2)	0.31	71 164 (62.4)	2431 (63.4)	0.36	5 690 762 (55.0)	254 186 (62.1)	0.51
Supervision in intensive phase^c^	3 480 789 (33.2)	114 754 (27.7)	0.43	6916 (28.2)	148 (25.3)	0.21	24 811 (21.7)	829 (21.6)	0.39	3 449 062 (33.3)	113 777 (27.8)	0.43
Full-course management^d^	1 099 121 (10.5)	37 244 (9.0)	0.41	10 266 (41.8)	221 (37.8)	0.22	15 787 (13.8)	501 (13.1)	0.38	1 073 068 (10.4)	36 522 (8.9)	0.41
Self-medication^e^	135 141 (1.3)	5162 (1.2)	0.46	2081 (8.5)	56 (9.6)	0.24	2318 (2.0)	73 (1.9)	0.37	130 742 (1.3)	5033 (1.2)	0.47
Case-finding pattern												
Direct visit to TB-designated facility	4 341 306 (41.4)	166 473 (40.2)	0.42	12 678 (51.6)	285 (48.8)	0.20	46 820 (41.0)	1648 (43.0)	0.36	4 281 808 (41.4)	164 540 (40.2)	0.43
Referral by non–TB-designated facility	4 505 800 (43.0)	181 607 (43.9)	0.48	7054 (28.7)	176 (30.1)	0.23	50 832 (44.6)	1679 (43.8)	0.35	4 447 914 (43.0)	179 752 (43.9)	0.48
Active screening	268 096 (2.6)	10 339 (2.5)	0.58	1257 (5.1)	22 (3.8)	0.17	1481 (1.3)	50 (1.3)	0.41	265 358 (2.6)	10 267 (2.5)	0.59
Tracing by NTP	1 367 069 (13.0)	55 517 (13.4)	0.68	3568 (14.5)	101 (17.3)	0.65	14 947 (13.1)	457 (11.9)	0.48	1 348 554 (13.0)	54 959 (13.4)	0.68
Second-month SS finding												
Positive	165 300 (1.6)	9678 (2.3)	0.67	74 (0.3)	11 (1.9)	1.59	1223 (1.1)	69 (1.8)	0.58	164 003 (1.6)	9598 (2.3)	0.68
Negative	9 942 636 (94.9)	390 005 (94.2)	0.47	15 996 (65.1)	405 (69.3)	0.27	107 459 (94.2)	3608 (94.1)	0.37	9 819 181 (94.9)	385 992 (94.3)	0.47
Unknown	374 335 (3.6)	14 253 (3.4)	0.43	8487 (34.6)	168 (28.8)	0.17	5398 (4.7)	157 (4.1)	0.34	360 450 (3.5)	13 928 (3.4)	0.43
Fifth-month SS finding												
Negative	10 394 (0.1)	611 (0.1)	0.77	12 (0.05)	0	0	88 (0.1)	9 (0.2)	1.12	10 294 (0.1)	602 (0.1)	0.76
Positive	9 699 099 (92.5)	384 533 (92.9)	0.48	14 006 (57.0)	364 (62.3)	0.28	103 125 (90.4)	3467 (90.4)	0.37	9 581 968 (92.6)	380 702 (93.0)	0.48
Unknown	772 778 (7.4)	28 792 (7.0)	0.39	10 539 (42.9)	220 (37.7)	0.19	10 867 (9.5)	358 (9.3)	0.35	751 372 (7.3)	28 214 (6.9)	0.39
Patient delay, wk												
<1	2 428 915 (23.2)	88 281 (21.3)	0.47	5911 (24.1)	142 (24.3)	0.28	19 314 (16.9)	619 (16.1)	0.39	2 403 690 (23.2)	87 520 (21.4)	0.48
1-4	2 943 475 (28.1)	109 086 (26.4)	0.47	6394 (26.0)	129 (22.1)	0.22	29 594 (25.9)	915 (23.9)	0.36	2 907 487 (28.1)	108 042 (26.4)	0.47
>4	5 109 881 (48.7)	216 569 (52.3)	0.48	12 252 (49.9)	313 (53.6)	0.23	65 172 (57.1)	2300 (60.0)	0.36	5 032 457 (48.7)	213 956 (52.2)	0.48
Diagnostic delay, wk												
≤1	7 942 042 (75.8)	315 624 (76.2)	0.48	18 400 (74.9)	429 (73.5)	0.25	86 445 (75.8)	2919 (76.1)	0.38	7 837 197 (75.8)	312 276 (76.3)	0.48
>1	2 540 229 (24.2)	98 312 (23.8)	0.45	6157 (25.1)	155 (26.5)	0.22	27 635 (24.2)	915 (23.9)	0.34	2 506 437 (24.2)	97 242 (23.7)	0.45
HIV infection												
Positive	33 681 (0.3)	1285 (0.3)	0.67	52 (0.2)	2 (0.3)	0.64	1714 (1.5)	37 (1.0)	0.40	31 915 (0.3)	1246 (0.3)	0.68
Negative or unknown	10 448 590 (99.7)	412 651 (99.7)	0.47	24 505 (99.8)	582 (99.7)	0.24	112 366 (98.5)	3797 (99.0)	0.37	10 311 719 (99.7)	408 272 (99.7)	0.48
Treatment outcome												
Cured	4 507 907 (43.0)	202 119 (48.8)	0.49	3046 (12.4)	111 (19.0)	0.35	35 814 (31.4)	1351 (35.2)	0.38	4 469 047 (43.2)	200 657 (49.0)	0.49
Treatment completed	5 974 364 (57.0)	211 817 (51.2)	0.46	21 511 (87.6)	473 (81.0)	0.22	78 266 (68.6)	2483 (64.8)	0.36	5 874 587 (56.8)	208 861 (51.0)	0.47

Abbreviations: 2HRZE/4HR, 2 months of isoniazid, rifampicin, pyrazinamide, and ethambutol followed by 4 months of isoniazid and rifampicin; 2HRZE/7-10HRE, 2 months of isoniazid, rifampicin, pyrazinamide, and ethambutol followed by 7 to 10 months of isoniazid, rifampicin, and ethambutol; 2HRZE/10HRE, 2 months of isoniazid, rifampicin, pyrazinamide, and ethambutol followed by 10 months of isoniazid, rifampicin, and ethambutol; MDR, multidrug resistant; NTP, national tuberculosis (TB) program; PTB, pulmonary TB; PY, person-years; SS, sputum smear.

^a^ Includes all 55 non-Han ethnic groups.

^b^ Patients take all doses under the direct observation of medication supervisors during the full course of treatment. Full-course supervision should be conducted for patients with SS–positive TB and new hematogenous disseminated and/or cavitary SS-negative TB.

^c^ Patients take all doses under the direct observation of medication supervisors during the intensive phase. Full-course management is conducted during the continuation phase. Supervision in intensive phase should be conducted for new non–hematogenous disseminated and/or noncavitary SS-negative TB and TB pleuritis.

^d^ Comprehensive management is conducted for patients during the full course of treatment to ensure medication is taken as prescribed, including health education, regular drug collection, family visit, check of medication (eg, pill count and urine test), and tracing in the case of failure to visit clinics or collect drugs.

^e^ Although health education on standardized chemotherapy has been provided, patients self-medicate due to lack of effective management.

**Figure 1.**
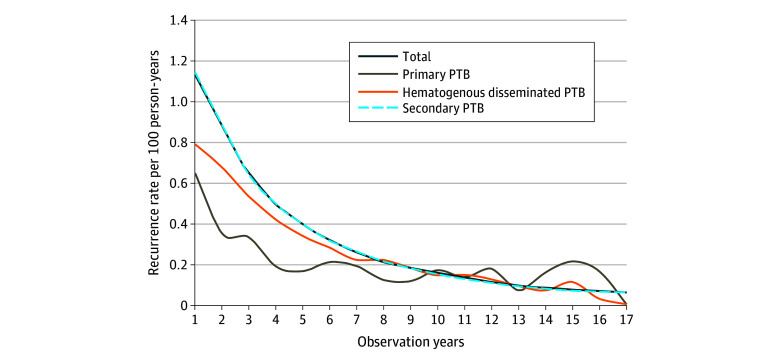
Trend of Pulmonary Tuberculosis (PTB) Recurrence Rate in China, 2005 to 2021 Recurrence rates are calculated by dividing the number of individuals with recurrence by observed person-years in year *n*.

Among the 10 482 271 patients with newly diagnosed PTB and successful treatment outcomes, 413 936 developed a second TB episode, resulting in an overall recurrence proportion of 3.9% (Table 1) and an overall recurrence rate of 0.47 (95% CI, 0.47-0.48) per 100 person-years. The recurrence rate decreased from 1.14 (95% CI, 1.14-1.15) per 100 person-years in the first year after enrollment to 0.06 (95% CI, 0.04-0.08) per 100 person-years in the 17th year (Figure 1 and eTable 1 in Supplement 1). The 2-year recurrence rate was 1.02 (95% CI, 1.01-1.02) per 100 person-years, and the 5-year recurrence rate was 0.74 (95% CI, 0.73-0.74) per 100 person-years. Nearly half (48.9%) of patients with recurrent TB experienced a recurrence within the first 2 years after completing primary treatment (28.2% in the first year and 20.7% in the second year). The proportion of patients with recurrent TB remained above 1.00% per year until year 11 after enrollment (eTable 2 in Supplement 1).

**Figure 2.**
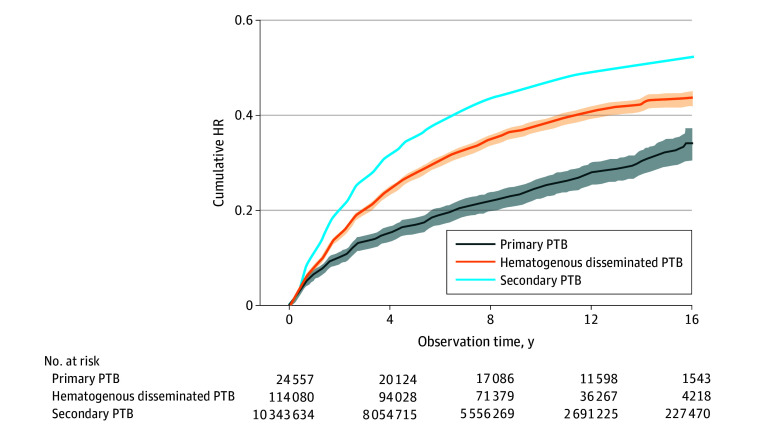
Cumulative Hazard of Recurrent Pulmonary Tuberculosis (PTB) in China, Disaggregated by Classification, 2005 to 2021 HR indicates hazard ratio. The shaded areas represent 95% CIs.

Patients aged 45 to 64 years accounted for 38.5% of all recurrent cases. The overall recurrence rate of primary PTB was 0.24 (95% CI, 0.22-0.26) per 100 person-years; hematogenous disseminated PTB, 0.37 (95% CI, 0.36-0.38) per 100 person-years; and secondary PTB, 0.48 (95% CI, 0.47-0.48) per 100 person-years. In most subgroups, secondary PTB was associated with a higher recurrence rate than primary PTB or hematogenous disseminated PTB. However, in the group with positive results of a sputum smear in the second month, those patients with primary TB had the highest recurrence rate (Table 1 and eTable 3 in Supplement 1). The cumulative risk of recurrence for all 3 disease categories varied significantly by sex, age, and occupation (eFigure 2 in Supplement 1). The difference in cumulative recurrence probability was greatest in the fifth year of observation and remained stable in subsequent years (Figure 2).

We calculated recurrence rates stratified by baseline characteristics to allow for direct comparisons (Table 1) and adjusted the HRs to control for confounding (Table 2). In the primary PTB cohorts, the adjusted HRs (AHRs) for recurrent TB were associated with an increased risk of recurrence among patients who were aged 65 to 84 years (AHR, 2.54 [95% CI, 1.65-3.92]), belonged to an ethnic minority group (AHR, 1.55 [95% CI, 1.25-1.91]), received treatment at a general hospital (AHR, 2.35 [95% CI, 1.26-4.41]), were identified through tracing (AHR, 1.44 [95% CI, 1.11-1.85]), while negative findings of bacteriological testing and second-month sputum smear and rifampin sensitivity were protective. In the hematogenous disseminated PTB cohorts, minority ethnicity (AHR, 1.40 [95% CI, 1.29-1.51]), migrant status (AHR, 1.34 [95% CI, 1.20-1.50]), treatment at a general hospital (AHR, 1.36 [95% CI, 1.12-1.66]), and intensive phase supervision (AHR, 1.12 [95% CI, 1.03-1.22]) were associated with an increased risk of recurrence, while female sex, negative findings of bacteriological testing, second- and fifth-month sputum smears, and rifampin sensitivity were protective. In the secondary PTB cohort, 15 to 24 years of age (AHR, 1.38 [95% CI, 1.28-1.49]), minority ethnicity (AHR, 1.76 [95% CI, 1.74-1.77]), agricultural work (AHR, 1.14 [95% CI, 1.12-1.16]), commercial work (AHR, 1.05 [95% CI, 1.02-1.09]), retired status (AHR, 1.06 [95% CI, 1.04-1.09]), unemployed status or being a homemaker (AHR, 1.32 [95% CI, 1.29-1.35]), migrant status (AHR, 1.13 [95% CI, 1.12-1.15]), treatment at a general hospital (AHR, 1.06 [95% CI, 1.04-1.07]), and identification through close contact tracing (AHR, 1.23 [95% CI, 1.22-1.24]) were associated with an increased risk of recurrence, while female sex, negative findings of bacteriological testing and second- and fifth-month sputum smears, and rifampin sensitivity were protective (Table 2).

**Table 2.** Multivariable Analysis of Risk Factors Associated With Recurrence Among Patients With Newly Diagnosed PTB in China, 2005 to 2021

Characteristic	AHR (95% CI)			
	Total	Primary PTB	Hematogenous disseminated PTB	Secondary PTB
Sex				
Male	1 [Reference]	1 [Reference]	1 [Reference]	1 [Reference]
Female	0.81 (0.80-0.82)^a^	1.00 (0.85-1.18)	0.75 (0.70-0.81)^a^	0.81 (0.80-0.82)^a^
Age, y				
0-4	0.19 (0.13-0.29)^a^	0.32 (0.16-0.64)^b^	0.79 (0.29-2.17)	0.12 (0.06-0.21)^a^
5-14	1 [Reference]	1 [Reference]	1 [Reference]	1 [Reference]
15-24	1.46 (1.36-1.56)^a^	1.64 (1.27-2.11)^a^	1.53 (1.09-2.16)^a^	1.38 (1.28-1.49)^a^
25-44	1.29 (1.20-1.38)^a^	1.18 (0.80-1.73)	1.34 (0.94-1.91)	1.22 (1.13-1.32)^a^
45-64	1.77 (1.65-1.89)^a^	2.29 (1.57-3.34)^a^	1.61 (1.13-2.29)^b^	1.68 (1.56-1.81)^a^
65-84	1.56 (1.45-1.67)^a^	2.54 (1.65-3.92)^a^	1.28 (0.89-1.83)	1.48 (1.37-1.59)^a^
≥85	0.89 (0.82-0.96)^b^	IC	0.84 (0.45-1.59)	0.84 (0.77-0.92)^a^
Ethnicity				
Han	1 [Reference]	1 [Reference]	1 [Reference]	1 [Reference]
Ethnic minority group^c^	1.75 (1.73-1.76)^a^	1.55 (1.25-1.91)^a^	1.40 (1.29-1.51)^a^	1.76 (1.74-1.77)^a^
Unknown	0.69 (0.57-0.84)^a^	IC	3.10 (0.77-12.46)	0.68 (0.56-0.83)^a^
Occupation				
Student	1 [Reference]	1 [Reference]	1 [Reference]	1 [Reference]
Preschooler	0.84 (0.67-1.06)	0.83 (0.50-1.39)	0.55 (0.22-1.38)	0.88 (0.67-1.16)
Industrial worker	1.01 (0.99-1.03)	0.98 (0.54-1.78)	0.79 (0.63-1.00)	1.01 (0.99-1.04)
Agricultural worker	1.14 (1.11-1.16)^a^	0.85 (0.63-1.13)	0.90 (0.77-1.04)	1.14 (1.12-1.16)^a^
Commercial worker	1.05 (1.02-1.08)^b^	1.05 (0.38-2.91)	1.02 (0.73-1.43)	1.05 (1.02-1.09)^b^
Health care worker	0.79 (0.74-0.85)^a^	1.06 (0.26-4.37)	0.77 (0.34-1.75)	0.79 (0.74-0.85)^a^
Retired	1.06 (1.03-1.09)^a^	1.52 (0.87-2.67)	0.93 (0.70-1.24)	1.06 (1.04-1.09)^a^
Office worker	0.87 (0.85-0.90)^a^	0.51 (0.18-1.43)	0.80 (0.57-1.14)	0.88 (0.85-0.90)^a^
Domestic duties or unemployed	1.31 (1.29-1.34)^a^	1.11 (0.68-1.81)	0.89 (0.73-1.08)	1.32 (1.29-1.35)^a^
Other	1.06 (1.04-1.09)^a^	0.90 (0.55-1.47)	0.80 (0.64-1.01)	1.07 (1.04-1.09)^a^
Migrant				
No	1 [Reference]	1 [Reference]	1 [Reference]	1 [Reference]
Yes	1.13 (1.12-1.15)^a^	1.35 (0.92-1.97)	1.34 (1.20-1.50)^a^	1.13 (1.12-1.15)^a^
Bacteriological test result				
Positive	1 [Reference]	1 [Reference]	1 [Reference]	1 [Reference]
Negative	0.84 (0.82-0.86)^a^	0.66 (0.43-0.99)^a^	0.75 (0.63-0.89)^b^	0.85 (0.83-0.86)^a^
Unknown	0.81 (0.76-0.85)^a^	0.61 (0.38-0.98)^a^	0.93 (0.65-1.33)	0.82 (0.78-0.87)^a^
Rifampin resistance				
Resistant	1 [Reference]	1 [Reference]	1 [Reference]	1 [Reference]
Sensitive	0.46 (0.42-0.51)^a^	0.08 (0.01-0.63)^a^	0.19 (0.05-0.76)^a^	0.47 (0.42-0.52)^a^
Unknown	0.26 (0.24-0.29)^a^	0.03 (0.01-0.25)^b^	0.12 (0.03-0.49)^b^	0.26 (0.24-0.29)^a^
Therapeutic regimen				
2HRZE/4HR	1 [Reference]	1 [Reference]	1 [Reference]	1 [Reference]
2HRZE/7-10HRE	0.96 (0.92-1.00)^a^	0.69 (0.32-1.51)	0.87 (0.60-1.26)	0.96 (0.92-1.00)
2HRZE/10HRE	0.84 (0.80-0.87)^a^	0.87 (0.21-3.52)	0.90 (0.67-1.21)	0.84 (0.81-0.87)^a^
Personalized	0.92 (0.90-0.93)^a^	0.79 (0.61-1.02)	0.83 (0.72-0.96)^a^	0.92 (0.91-0.93)^a^
MDR	0.67 (0.61-0.73)^a^	IC	0.93 (0.23-3.74)	0.66 (0.60-0.73)^a^
Treatment management institution				
Infectious disease hospital	1 [Reference]	1 [Reference]	1 [Reference]	1 [Reference]
Primary health institution	0.98 (0.94-1.02)	1.12 (0.35-3.56)	1.45 (0.88-2.39)	0.98 (0.93-1.02)
Disease control institution	0.83 (0.82-0.84)^a^	1.14 (0.62-2.08)	1.10 (0.91-1.33)	0.83 (0.82-0.84)^a^
General hospital	1.06 (1.05-1.08)^a^	2.35 (1.26-4.41)^b^	1.36 (1.12-1.66)^b^	1.06 (1.04-1.07)^a^
Supervision patterns				
Full-course supervision^d^	1 [Reference]	1 [Reference]	1 [Reference]	1 [Reference]
Supervision in intensive phase^e^	0.83 (0.82-0.84)^a^	0.91 (0.70-1.18)	1.12 (1.03-1.22)^a^	0.82 (0.82-0.83)^a^
Full-course management^f^	0.80 (0.79-0.81)^a^	1.03 (0.81-1.31)	1.02 (0.93-1.13)	0.79 (0.78-0.80)^a^
Self-medication^g^	0.95 (0.92-0.98)^a^	1.25 (0.89-1.76)	0.97 (0.77-1.23)	0.95 (0.92-0.97)^a^
Case-finding pattern				
Direct visit to TB-designated facility	1 [Reference]	1 [Reference]	1 [Reference]	1 [Reference]
Referral by non–TB-designated facility	1.05 (1.05-1.06)^a^	1.04 (0.86-1.26)	0.96 (0.90-1.03)	1.05 (1.05-1.06)^a^
Active screening	1.11 (1.09-1.14)^a^	0.85 (0.54-1.32)	1.03 (0.78-1.37)	1.11 (1.09-1.13)^a^
Tracing by NTP	1.23 (1.22-1.24)^a^	1.44 (1.11-1.85)^b^	1.05 (0.95-1.17)	1.23 (1.22-1.24)^a^
Second-month sputum smear finding				
Positive	1 [Reference]	1 [Reference]	1 [Reference]	1 [Reference]
Negative	0.85 (0.83-0.87)^a^	0.31 (0.16-0.58)^a^	0.72 (0.56-0.91)^b^	0.85 (0.83-0.87)^a^
Unknown	0.97 (0.94-0.99)^a^	0.36 (0.18-0.71)^b^	0.68 (0.50-0.92)^a^	0.97 (0.94-1.00)^a^
Fifth-month sputum smear finding				
Positive	1 [Reference]	1 [Reference]	1 [Reference]	1 [Reference]
Negative	0.78 (0.72-0.84)^a^	IC	0.33 (0.17-0.64)^a^	0.78 (0.72-0.85)^a^
Unknown	0.74 (0.68-0.80)^a^	IC	0.35 (0.18-0.69)^b^	0.74 (0.68-0.80)^a^
Patient delay, wk				
<1	1 [Reference]	1 [Reference]	1 [Reference]	1 [Reference]
1-4	0.99 (0.98-1.00)	0.87 (0.68-1.11)	0.95 (0.85-1.05)	0.99 (0.99-1.00)
>4	1.06 (1.05-1.07)^a^	1.06 (0.86-1.30)	1.03 (0.94-1.13)	1.06 (1.05-1.07)^a^
Diagnostic delay, wk				
≤1	1 [Reference]	1 [Reference]	1 [Reference]	1 [Reference]
>1	0.98 (0.98-0.99)^a^	1.07 (0.88-1.30)	1.01 (0.93-1.08)	0.98 (0.98-0.99)^a^
HIV infection				
Positive	1 [Reference]	1 [Reference]	1 [Reference]	1 [Reference]
Negative or unknown	1.03 (0.97-1.09)	0.61 (0.15-2.51)	1.31 (0.94-1.81)	1.01 (0.95-1.07)
Treatment outcome				
Cured	1 [Reference]	1 [Reference]	1 [Reference]	1 [Reference]
Treatment completed	1.16 (1.14-1.18)^a^	1.30 (0.86-1.96)	1.13 (0.95-1.35)	1.16 (1.14-1.19)^a^

Abbreviations: 2HRZE/4HR, 2 months of isoniazid, rifampicin, pyrazinamide, and ethambutol followed by 4 months of isoniazid and rifampicin; 2HRZE/7-10HRE, 2 months of isoniazid, rifampicin, pyrazinamide, and ethambutol followed by 7 to 10 months of isoniazid, rifampicin, and ethambutol; 2HRZE/10HRE, 2 months of isoniazid, rifampicin, pyrazinamide, and ethambutol followed by 10 months of isoniazid, rifampicin, and ethambutol; AHR, adjusted hazard ratio; IC, incapable coefficient; MDR, multidrug resistant; NTP, national tuberculosis (TB) program; PTB, pulmonary TB.

^a^ *P* < .001.

^b^ *P* < .01.

^c^ Includes all 55 non-Han ethnic groups.

^d^ Patients take all doses under the direct observation of medication supervisors during the full course of treatment. Full-course supervision should be conducted for patients with sputum smear (SS)–positive TB and new hematogenous disseminated and/or cavitary SS-negative TB.

^e^ Patients take all doses under the direct observation of medication supervisors during the intensive phase. Full-course management is conducted during the continuation phase. Supervision in intensive phase should be conducted for new non–hematogenous disseminated and/or noncavitary SS-negative TB and TB pleuritis.

^f^ Comprehensive management is conducted for patients during the full course of treatment to ensure medication is taken as prescribed, including health education, regular drug collection, family visit, check of medication (eg, pill count and urine test), and tracing in the case of failure to visit clinics or collect drugs.

^g^ Although health education on standardized chemotherapy has been provided, patients self-medicate due to lack of effective management.

**Figure 3.**
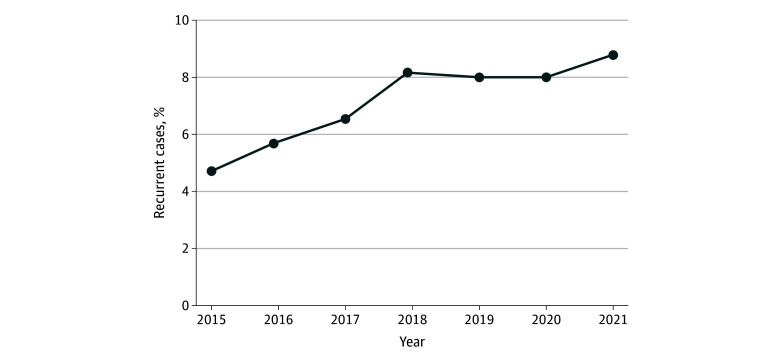
Proportions of Recurrent Cases Among Incident Cases of Pulmonary Tuberculosis in China, 2015 to 2021

The proportion of recurrent cases among reported incident cases increased 1.9-fold from 4.7% in 2015 to 8.8% in 2021 (Figure 3). The 1- and 2-year recurrence rates generally trended upward for all notification years. The 1-year recurrence rate increased from 0.82 (95% CI, 0.79-0.86) per 100 person-years in 2005 to 2.18 (95% CI, 2.14-2.22) per 100 person-years in 2018 and then began to decline to 1.73 (95% CI, 1.69-1.77) per 100 person-years in 2020. The 2-year recurrence rate also increased from 0.72 (95% CI, 0.70-0.75) per 100 person-years in 2005 to 1.87 (95% CI, 1.85-1.89) per 100 person-years in 2017 and then declined to 1.52 (95% C, 1.50-1.54) per 100 person-years in 2019 (eFigure 3 in Supplement 1).

Recurrence rates varied substantially across provinces, ranging from 0.26 (95% CI, 0.26-0.27) per 100 person-years in Guangxi to 1.42 (95% CI, 1.40-1.43) per 100 person-years in Xinjiang (eFigure 4 in Supplement 1). Most recurrent episodes were classified as secondary PTB. Some patients with primary PTB (12.7%) represented a primary syndrome when they developed recurrent TB. Few patients with PTB developed recurrent extrapulmonary TB (eTable 4 in Supplement 1).

## Discussion

This cohort study provides a systematic and comprehensive overview of the PTB recurrence rate based on analysis of what is to our knowledge the largest scale surveillance cohort in the world. The results provide robust and important evidence to inform policy-making on global TB elimination.

This study found that the mean recurrence risk among patients with newly diagnosed PTB in China was 8.5 times higher than the estimated TB incidence (55 per 100 000) in 2021.^21^ The recurrence probability was not evenly distributed throughout the entire posttreatment period. Recurrence within 2 years has been an important indicator of TB treatment effectiveness.^22^ Our results showed that the 1- and 2-year recurrence rates were high (1.14 and 0.89 per 100 person-years, respectively), and these early recurrences accounted for almost half (48.9%) of all recurrent cases. This finding highlights a window of opportunity for potential interventions. Dodd et al^23^ estimated there were 155 million individuals who survived TB worldwide in 2020, 8% of whom received treatment in the previous 2 years. Continuous posttreatment follow-up with active screening could facilitate timely detection of recurrence. Isoniazid secondary preventive treatment has also been shown to be an effective intervention for reducing recurrence; however, evidence supporting its use has been primarily limited to patients with TB who are positive for HIV.^24,25^

The increasing trend of recurrent cases among reported incident cases also highlights the urgent need to address TB recurrence. In this study, the proportion of incident cases that were recurrent increased from 4.7% in 2015 to 8.8% in 2021. Globally, 5.5% of incident TB cases were recurrent, and this exceeded 20% in certain countries, such as Kazakhstan and the Russia Federation.^5^ This study also found that the 1- and 2-year recurrence rates have increased substantially since 2005 (eFigure 3 in Supplement 1). The burden of recurrent TB is likely to pose a growing challenge in China and globally. Although most recurrences occurred in the early posttreatment period, long-term recurrence should not be overlooked. Most previous studies of TB recurrence have had short observation periods. A 2021 systematic review by Vega et al^6^ of global TB recurrence studies showed that the mean observation time in these studies was 2.3 years. The electronic cohort used in the present study was based on data from a national surveillance system that captures approximately 92% of patients diagnosed with TB annually.^16^ The use of national data also mitigates the underestimate of recurrence that can result from patient migration between cities or loss to follow-up. In this study, even with a follow-up period exceeding 11 years, 1% of patients with a history of TB still experienced a recurrent episode each year. Digital technologies may provide a more convenient and effective approach to long-term follow-up.^26^

This study found that the TB recurrence rate was associated with numerous factors. Consistent with previous studies,^11,27,28,29,30^ male sex, minority ethnicity, unemployment, migrant status, and positive findings on bacteriological testing were associated with an increased likelihood of recurrent TB. More than one-third of patients who experienced recurrence were aged 45 to 64 years at the time of their initial PTB diagnosis. This finding is consistent with another regional study in Tianjin^30^ but contrasts with findings from Rio de Janeiro, Brazil.^31^ In that study, patients treated at disease control centers had a lower risk of recurrence. In China, disease control centers are fully government-funded, not-for-profit institutions. In contrast, general hospitals and other health institutions are typically only partially government funded and have a high clinical workload, which may interfere with their public health duties and lead to less effective TB reporting^16^ and management.^32^ For secondary PTB, patients classified as having completed treatment had a higher recurrence risk than those classified as cured. This finding is consistent with that of Cox et al in Uzbekistan^33^ and could be attributed to the lack of last sputum smear results after completion of full-course treatment. Consistent with a previous study from Henan province,^34^ patients with rifampin resistance had a higher risk of recurrence than those with drug-susceptible TB. However, the risk of recurrence was greatly reduced among patients who received an appropriate regimen for drug-resistant TB. In the stratified analysis, the recurrence risk of patients with rifampin resistance who received an MDR regimen was only 0.27 times that of patients who received the standardized regimen for drug-susceptible TB (eTable 5 in Supplement 1), highlighting the importance of drug susceptibility testing prior to treatment initiation. Meanwhile, for primary and hematogenous disseminated PTB with no evidence of drug resistance, there was no significant difference in efficacy between treatment regimens. Prolonged use of drugs may not result in a better therapeutic effect. The WHO announced changes to shorten the duration of drug-susceptible TB treatment in its updated consolidated guidelines. These shorter, more effective treatment regimens improve quality of life for patients, reduce their duration of illness, and reduce the burden on health systems.^35^

The emergence of TB recurrence necessitates the exploration of new strategies to reduce this risk. The use of appropriate regimens for different patient populations, especially those with drug-resistant TB, is the most reliable method to reduce endogenous reactivation. This requires not only identifying all potential drug-resistant cases but also ensuring that patients have access to appropriate second-line regimens given the current low coverage of drug-resistant TB treatment.^36^ Meanwhile, regardless of whether endogenous reactivation or exogenous reinfection has occurred, early diagnosis is crucial to prevent unfavorable outcomes and reduce potential community transmission. Using a transmission-dynamic mathematical model combined with cost-effectiveness analysis, Marx et al^37,38^ evaluated different strategies for annual posttreatment observation and secondary isoniazid preventive therapy and found that a single observation examination at the end of the first year after treatment completion combined with 12 months of secondary isoniazid preventive therapy can accelerate reductions in TB incidence and reduce the cost of TB control in a setting with a high TB incidence. Interventions for recurrent TB should be prioritized for patients who have recently completed TB treatment to improve their quality of life and prevent them from experiencing another TB episode.^23^

### Limitations

This study has several limitations. First, we did not have data that would allow us to determine the underlying cause of recurrence (reactivation vs reinfection). Two systematic reviews^6,39^ indicated that reactivation may be the main reason for recurrence in most settings, consistent with previous studies in China.^40,41,42^ However, studies in South Africa^43,44^ showed reinfection could also dominate in areas with a very high TB burden and that the risk of reinfection can persist for many years. Second, although HIV infection is a known risk factor for recurrent TB,^45^ it was not identified as a significant risk factor for any of the 3 types of TB in this study. However, China has a low HIV burden, with an estimated HIV-positive TB incidence rate of only 0.93 per 100 000 population.^21^ Given the high dimensionality of covariates included in our model, it is possible that HIV infection was not a significant risk factor because the variance of the outcome was explained by the combination of the other covariates.

## Conclusions

The findings of this cohort study suggest that there is a high burden of recurrent TB in China, and this is likely to be an even greater challenge in the future. Patients with successful TB treatment history have a much higher risk of developing another new episode than the general population, with almost half of recurrences occurring within the first 2 years post treatment. These findings suggest that routine posttreatment follow-up should be considered as a strategy to accelerate TB elimination efforts in China.

## Notes

Supplement 1. **eAppendix.** Probabilistic Matching Algorithm **eTable 1.** Trend of PTB Recurrence Rate in China, 2005 to 2021 **eTable 2.** Proportions of Recurrence Among Patients With PTB Aggregated by Follow-Up Years in China **eTable 3.** Adjusted Hazard Ratio of Recurrence Compared Among Primary, Hematogenous Disseminated, and Secondary PTB in China, 2005 to 2021 **eTable 4.** TB Classifications: First Episode vs Recurrent Episode **eTable 5.** Risk of Disease Classification, Drug Resistance, and Therapy Regimens **eFigure 1.** Group Process of Study **eFigure 2.** Cumulative Hazard of Recurrent PTB in China, Disaggregated by Subgroups, 2005 to 2021 **eFigure 3.** Recurrence Rate of i Year **eFigure 4.** Recurrence Rates in Different Provinces of China, 2005 to 2021 **eFigure 5.** Schoenfeld Tests for Cox Proportional Risk Assumption

Supplement 2.
Data Sharing Statement
