# Supplementary material for: Recurrent Pulmonary Tuberculosis in China, 2005 to 2021
Source: JAMA Netw Open. 2024 Aug 12;7(8):e2427266. doi: 10.1001/jamanetworkopen.2024.27266 (PMC11320166; doi:10.1001/jamanetworkopen.2024.27266)
Supplement: Supplement 1. — eAppendix. Probabilistic Matching Algorithm eTable 1. Trend of PTB Recurrence Rate in China, 2005 to 2021 eTable 2. Proportions of Recurrence Among Patients With PTB Aggregated by Follow-Up Years in China eTable 3. Adjusted Hazard Ratio of Recurrence Compared Among Primary, Hematogenous Disseminated, and Secondary PTB in China, 2005 to 2021 eTable 4. TB Classifications: First Episode vs Recurrent Episode eTable 5. Risk of Disease Classification, Drug Resistance, and Therapy Regimens eFigure 1. Group Process of Study eFigure 2. Cumulative Hazard of Recurrent PTB in China, Disaggregated by Subgroups, 2005 to 2021 eFigure 3. Recurrence Rate of i Year eFigure 4. Recurrence Rates in Different Provinces of China, 2005 to 2021 eFigure 5. Schoenfeld Tests for Cox Proportional Risk Assumption [file jamanetwopen-e2427266-s001.pdf]

Supplementary Online Content

Li T, Zhang B, Du X, Pei S, Jia Z, Zhao Y. Recurrent pulmonary tuberculosis in China, 2005 to 2021. *JAMA Netw Open*. 2024;7(8):e2427266. doi:10.1001/jamanetworkopen.2024.27266

eAppendix. Probabilistic Matching Algorithm
eTable 1. Trend of PTB Recurrence Rate in China, 2005 to 2021
eTable 2. Proportions of Recurrence Among Patients With PTB Aggregated by Follow-Up Years in China
eTable 3. Adjusted Hazard Ratio of Recurrence Compared Among Primary, Hematogenous Disseminated, and Secondary PTB in China, 2005 to 2021
eTable 4. TB Classifications: First Episode vs Recurrent Episode
eTable 5. Risk of Disease Classification, Drug Resistance, and Therapy Regimens
eFigure 1. Group Process of Study
eFigure 2. Cumulative Hazard of Recurrent PTB in China, Disaggregated by Subgroups, 2005 to 2021
eFigure 3. Recurrence Rate of i Year
eFigure 4. Recurrence Rates in Different Provinces of China, 2005 to 2021
eFigure 5. Schoenfeld Tests for Cox Proportional Risk Assumption

This supplementary material has been provided by the authors to give readers additional information about their work.

eAppendix. Probabilistic Matching Algorithm

1. Make records with unique identifiers (ID card number/passport number) the gold standard; Records without unique identifiers are taken as the data set to be matched (Attached Tables), and "name + date of birth + domicile code" is used for probability matching.

2. The two records are matched. First, the similarity of the names is calculated using the text similarity algorithm (0~ 1,0 is completely different, 1 is completely consistent). Calculate the number of days between birth dates. If the number of days is greater than 365, the similarity score of birth date is 0; if the number is less than or equal to 365, the similarity score is (365-days between) /365, and the matching score is also 0 to 1. If the home code is exactly the same, the score is 0.2, otherwise it is 0. Therefore, the total score of the whole matching process ranges from 0 to 2.2. The specific score value used as the threshold for judging the matching needs to be verified in the fourth step.

3. Each record in the data set to be matched is matched with the gold standard and all other records in the data set to be matched. If a record in the gold standard is matched, the unique identifier of the gold standard is taken as the unique identifier of this record and all records it matches. If no record is matched in the gold standard, a unique identifier is dynamically generated as the unique identifier for this record and the matched record in the data set to be matched.

4. The effect of matching algorithm is compared based on sensitivity and specificity, and the gold standard is taken as reference. For a record, if there is a matching record in the gold standard and the matching algorithm matches, it is true positive (N1). If there is a matching record in the gold standard, but the matching algorithm matches incorrectly or does not match the record, it is false negative (N2+N3). If there is no matching record in the gold standard, and the matching algorithm matches the record, it is false positive (N4). If no matching record exists in the gold standard and the matching algorithm does not match the record, it is true negative (N5).

Matching results

Match result / Gold Criterion	Have ID		No ID
	Match	Not Match	
Have ID	N1	N2	N3
No ID	N4		N5

Sensitivity and Specificity of Different Threshold Value

**Threshold value**	**Sensitivity**	**Specificity**
1.96	85.1%	83.5%
1.97	86.3%	84.7%
1.98	87.2%	85.8%
1.99	89.6%	88.1%
**2.00**	**91.0%**	**90.0%**
2.01	88.4%	87.5%
2.02	85.7%	83.4%
2.03	83.9%	80.8%
2.04	81.6%	79.1%
2.05	79.8%	77.5%

© 2024 Li T et al. *JAMA Network Open.*

eTable 1. Trend of PTB Recurrence Rate in China, 2005 to 2021

Classification	Observation years																
	1	2	3	4	5	6	7	8	9	10	11	12	13	14	15	16	17
Total	1.14	0.89	0.64	0.49	0.39	0.31	0.26	0.21	0.18	0.15	0.13	0.11	0.09	0.08	0.07	0.06	0.06
Primary PTB	0.65	0.35	0.33	0.19	0.16	0.21	0.19	0.12	0.11	0.17	0.13	0.17	0.07	0.16	0.21	0.16	0.00
Hematogenous Disseminated PTB	0.79	0.68	0.53	0.42	0.34	0.28	0.22	0.22	0.18	0.14	0.14	0.12	0.09	0.07	0.11	0.03	0.00
Secondary PTB	1.15	0.89	0.64	0.49	0.39	0.31	0.26	0.21	0.18	0.15	0.13	0.11	0.09	0.08	0.07	0.06	0.06

Figure 1. Trend of PTB recurrence rate in China, 2005-2021

The number in each cell represents the recurrence rate, which was calculated by dividing the number of recurrent individuals by observed person years in year n

© 2024 Li T et al. *JAMA Network Open*.

eTable 2. Proportions of Recurrence Among Patients With PTB Aggregated by Follow-Up Years in China

Characteristics	Number	Observation years																
		1	2	3	4	5	6	7	8	9	10	11	12	13	14	15	16	17
**Total**	**413936**	**28.19**	**20.66**	**14.02**	**10.06**	**7.46**	**5.49**	**4.14**	**3.06**	**2.29**	**1.64**	**1.16**	**0.80**	**0.49**	**0.30**	**0.17**	**0.07**	**0.01**
Sex																		
Male	303486	28.59	20.98	14.06	9.99	7.31	5.41	4.05	2.98	2.24	1.56	1.12	0.75	0.46	0.29	0.15	0.07	0.01
Female	110450	27.09	19.79	13.92	10.27	7.87	5.71	4.37	3.26	2.44	1.84	1.29	0.92	0.58	0.34	0.21	0.08	0.01
Age																		
0~4	26	34.62	7.69	7.69	0.00	7.69	0.00	0.00	0.00	3.85	3.85	0.00	3.85	7.69	7.69	15.38	0.00	0.00
5~14	908	15.97	13.00	11.12	7.49	9.03	7.93	6.28	5.62	5.07	5.07	4.19	4.41	1.65	1.43	1.10	0.66	0.00
15~24	54337	26.29	18.04	12.95	9.90	7.67	6.24	5.01	3.79	3.13	2.43	1.74	1.22	0.74	0.44	0.29	0.10	0.01
25~44	107573	27.17	18.70	13.19	9.81	7.60	5.89	4.70	3.61	2.86	2.19	1.60	1.13	0.72	0.46	0.25	0.11	0.02
45~64	159429	27.40	20.95	14.21	10.40	7.66	5.66	4.20	3.12	2.24	1.49	1.07	0.72	0.43	0.26	0.13	0.06	0.00
65~84	89450	31.63	23.99	15.40	10.00	6.88	4.32	2.88	1.88	1.21	0.76	0.47	0.26	0.17	0.10	0.05	0.02	0.00
85~	2213	46.59	27.88	12.47	5.87	4.20	1.40	1.17	0.18	0.14	0.05	0.05	0.00	0.00	0.00	0.00	0.00	0.00
Ethnic																		
Han	350120	28.34	20.28	13.79	9.88	7.34	5.44	4.21	3.15	2.41	1.76	1.27	0.90	0.57	0.35	0.19	0.08	0.01
Minority	63719	27.34	22.74	15.28	11.04	8.10	5.73	3.75	2.55	1.65	0.95	0.57	0.24	0.06	0.01	0.00	0.00	0.00
Unknown	97	21.65	19.59	20.62	14.43	11.34	8.25	3.09	1.03	0.00	0.00	0.00	0.00	0.00	0.00	0.00	0.00	0.00
Occupation																		
Preschooler	78	25.64	17.95	11.54	6.41	7.69	7.69	3.85	5.13	1.28	3.85	1.28	1.28	1.28	2.56	2.56	0.00	0.00
Student	17008	25.62	16.84	12.92	9.64	7.93	6.46	5.06	3.89	3.31	2.90	2.00	1.48	0.81	0.59	0.40	0.15	0.01
Industrial worker	19197	25.53	17.93	13.03	9.61	7.79	6.11	5.29	4.25	3.28	2.63	1.78	1.23	0.77	0.44	0.24	0.09	0.01
Agricultural worker	293734	27.98	21.16	14.26	10.26	7.48	5.44	4.06	2.96	2.16	1.49	1.09	0.74	0.46	0.27	0.14	0.06	0.01
Commercial worker	5634	28.19	18.51	12.99	10.08	7.40	5.88	4.51	3.53	3.16	1.81	1.53	0.92	0.69	0.41	0.27	0.09	0.04
Healthcare worker	834	28.90	18.23	11.03	9.35	8.15	6.12	4.32	4.20	4.08	2.64	1.08	0.72	0.72	0.48	0.00	0.00	0.00

© 2024 Li T et al. *JAMA Network Open*.

Retired	13200	32.36	22.30	14.08	9.75	6.69	4.70	3.54	2.21	1.69	1.11	0.67	0.43	0.21	0.14	0.08	0.03	0.00
Office worker	6010	27.55	18.57	12.95	9.62	7.90	5.64	4.46	3.78	2.90	2.01	1.63	1.30	0.70	0.57	0.28	0.13	0.02
Domestic duties or unemployed	39685	30.95	21.26	13.97	9.48	7.11	5.10	3.65	2.66	2.00	1.46	0.93	0.62	0.39	0.23	0.12	0.06	0.00
Others	18556	27.95	18.14	13.04	9.48	7.50	5.97	4.63	3.61	3.00	2.26	1.61	1.10	0.75	0.49	0.32	0.12	0.03
Migrant																		
Yes	39052	26.39	18.59	13.38	10.67	8.57	7.02	5.41	4.22	2.70	1.53	0.87	0.38	0.16	0.07	0.04	0.02	0.00
No	374884	28.37	20.88	14.09	10.00	7.35	5.33	4.01	2.93	2.25	1.65	1.20	0.84	0.53	0.32	0.18	0.08	0.01
Bacteriologically Result																		
Positive	207474	30.22	20.41	12.93	9.24	6.93	5.23	4.15	3.17	2.47	1.81	1.29	0.90	0.58	0.36	0.20	0.09	0.01
Negative	204973	26.08	20.90	15.12	10.90	8.01	5.76	4.14	2.95	2.12	1.47	1.03	0.70	0.40	0.24	0.13	0.05	0.00
Unknown	1489	34.72	21.76	14.30	9.00	5.71	3.43	2.69	1.48	2.28	0.94	1.21	1.01	0.54	0.40	0.54	0.00	0.00
Rifampin Resistance																		
Resistance	422	55.69	23.93	10.43	4.50	2.61	1.42	0.71	0.47	0.24	0.00	0.00	0.00	0.00	0.00	0.00	0.00	0.00
Sensitive	33765	51.58	27.11	11.14	5.02	2.59	1.26	0.70	0.36	0.16	0.07	0.01	0.00	0.00	0.00	0.00	0.00	0.00
Unknown	379749	26.08	20.08	14.28	10.52	7.90	5.87	4.45	3.30	2.49	1.78	1.27	0.87	0.54	0.33	0.18	0.08	0.01
Therapeutic Regimen																		
2HRZE/4HR	383684	27.92	20.70	14.08	10.14	7.51	5.51	4.15	3.06	2.30	1.64	1.16	0.80	0.49	0.30	0.16	0.07	0.01
2HRZE/7-10HRE	2078	34.17	21.37	13.52	9.53	6.21	4.43	2.65	2.74	1.73	1.20	1.11	0.58	0.19	0.29	0.24	0.05	0.00
2HRZE/10HRE	2570	45.84	27.55	13.58	7.59	3.31	1.56	0.51	0.08	0.00	0.00	0.00	0.00	0.00	0.00	0.00	0.00	0.00
Personalized	25099	29.42	19.25	13.16	9.24	7.34	5.72	4.49	3.39	2.45	1.83	1.38	0.84	0.63	0.40	0.28	0.16	0.01
MDR	505	55.05	24.95	14.46	3.37	0.59	0.79	0.40	0.20	0.20	0.00	0.00	0.00	0.00	0.00	0.00	0.00	0.00
Treatment management institutions																		
Infectious disease hospital	25382	36.31	22.00	13.25	8.43	5.78	4.15	3.00	2.10	1.72	1.11	0.84	0.55	0.28	0.27	0.15	0.06	0.01

© 2024 Li T et al. *JAMA Network Open*.

Primary health institutions	2314	34.31	22.90	13.96	7.78	5.57	4.49	3.11	2.46	2.07	1.21	0.52	0.78	0.43	0.17	0.13	0.09	0.00
Disease control institutions	304505	24.65	19.18	13.98	10.62	8.26	6.35	4.89	3.67	2.75	1.98	1.42	0.97	0.60	0.36	0.20	0.09	0.01
General hospitals	81735	38.66	25.70	14.41	8.54	5.05	2.70	1.71	1.07	0.76	0.53	0.35	0.24	0.14	0.09	0.03	0.01	0.00
Supervision patterns																		
Full-course supervision	256776	29.09	20.69	13.58	9.68	7.17	5.31	4.07	3.09	2.36	1.71	1.24	0.85	0.54	0.34	0.19	0.08	0.01
Supervision in intensive phase	114754	26.07	20.71	15.03	11.02	8.20	5.95	4.34	2.94	2.14	1.43	0.96	0.63	0.31	0.17	0.08	0.01	0.00
Full-course management	37244	28.01	20.18	14.09	9.92	7.36	5.42	4.04	3.23	2.27	1.73	1.27	0.94	0.69	0.43	0.27	0.15	0.01
Self-medication	5162	31.44	21.46	12.73	8.62	6.32	4.67	3.64	2.81	2.42	2.03	1.45	1.10	0.60	0.25	0.25	0.19	0.00
Case finding pattern																		
Direct visit to TB designated facilities	166473	27.41	20.14	13.90	9.99	7.46	5.58	4.32	3.23	2.47	1.82	1.35	0.99	0.60	0.39	0.23	0.10	0.01
Referral by non-TB designated facilities	181607	27.59	20.45	14.10	10.21	7.55	5.54	4.20	3.13	2.37	1.72	1.22	0.82	0.54	0.32	0.16	0.07	0.01
Active screening	10339	32.95	25.94	12.22	7.91	5.78	4.41	3.23	2.39	1.87	1.27	0.78	0.64	0.36	0.16	0.05	0.04	0.00
Tracing by NTP	55517	31.57	21.94	14.45	10.18	7.48	5.25	3.56	2.43	1.59	0.88	0.47	0.18	0.01	0.00	0.00	0.00	0.00
2nd Month Sputum smear																		
Positive	9678	35.42	20.54	12.45	8.40	6.43	4.28	3.58	2.60	1.82	1.36	1.25	0.70	0.62	0.27	0.18	0.08	0.02
Negative	390005	27.93	20.67	14.10	10.16	7.51	5.53	4.17	3.07	2.30	1.63	1.15	0.78	0.47	0.29	0.15	0.06	0.01
Unknown	14253	30.32	20.56	12.85	8.34	6.78	5.23	3.52	2.89	2.33	1.92	1.53	1.33	0.98	0.68	0.47	0.26	0.01
5th Month Sputum smear																		
Negative	611	42.06	18.00	13.26	9.66	4.09	2.95	2.95	1.47	2.13	0.98	0.98	1.15	0.00	0.16	0.00	0.16	0.00
Positive	384533	28.11	20.74	14.13	10.17	7.51	5.49	4.15	3.05	2.27	1.59	1.11	0.76	0.45	0.27	0.14	0.06	0.01
Unknown	28792	28.92	19.69	12.50	8.62	6.86	5.58	3.99	3.21	2.67	2.23	1.88	1.36	1.04	0.73	0.47	0.24	0.01

© 2024 Li T et al. *JAMA Network Open*.

Patient Delay																		
≤1 week	88281	28.88	20.96	13.96	10.06	7.33	5.40	4.04	2.89	2.26	1.57	1.06	0.76	0.40	0.23	0.13	0.05	0.01
1~4 weeks	109086	28.77	20.86	14.37	10.22	7.34	5.39	4.01	2.94	2.17	1.47	1.04	0.66	0.36	0.22	0.11	0.05	0.01
＞4 weeks	216569	27.61	20.44	13.87	9.98	7.57	5.57	4.24	3.18	2.37	1.74	1.27	0.89	0.59	0.37	0.21	0.09	0.01
Diagnostic Delay																		
≤1 week	315624	28.36	20.70	14.08	10.11	7.47	5.50	4.12	3.07	2.27	1.59	1.13	0.74	0.43	0.24	0.13	0.05	0.01
＞1 week	98312	27.63	20.53	13.84	9.91	7.42	5.44	4.18	3.02	2.35	1.79	1.29	1.00	0.69	0.50	0.27	0.13	0.01
HIV Infection																		
Positive	1285	33.15	24.12	13.39	8.79	7.32	4.12	2.65	1.87	1.79	1.95	0.39	0.16	0.16	0.00	0.16	0.00	0.00
Negative or Unknown	412651	28.17	20.65	14.02	10.06	7.46	5.49	4.14	3.06	2.30	1.63	1.17	0.80	0.49	0.30	0.17	0.07	0.01
Treatment Outcome																		
Cured	202119	29.63	20.19	12.97	9.36	7.04	5.30	4.23	3.25	2.54	1.87	1.35	0.94	0.61	0.39	0.22	0.10	0.01
Treatment completed	211817	26.82	21.11	15.02	10.73	7.86	5.66	4.05	2.87	2.06	1.41	0.99	0.66	0.38	0.22	0.12	0.04	0.00

© 2024 Li T et al. *JAMA Network Open*.

eTable 3. Adjusted Hazard Ratio of Recurrence Compared Among Primary, Hematogenous Disseminated, and Secondary PTB in China, 2005 to 2021

Characteristic	Secondary PTB vs. Primary TB	Secondary PTB vs. Hematogenous Disseminated PTB	Hematogenous Disseminated PTB vs. Primary TB
**Total**	1.22(1.12-1.32) ***	1.27(1.23-1.31) ***	1.11(1.00-1.23)
Sex			
Male	1.19(1.07-1.33) **	1.22(1.17-1.26) ***	1.15(1.01-1.32) *
Female	1.23(1.08-1.40) **	1.42(1.34-1.50) ***	1.00(0.84-1.19)
Age			
0~4	1.45(0.59-3.54)	0.33(0.10-1.08)	3.81(1.30-11.21) *
5~14	1.47(1.19-1.82) ***	1.18(0.85-1.64)	1.16(0.77-1.76)
15~24	1.18(1.02-1.37) *	1.06(0.99-1.14)	1.31(1.08-1.58) **
25~44	1.61(1.30-2.01) ***	1.17(1.10-1.24) ***	1.42(1.13-1.80) **
45~64	1.13(0.93-1.37)	1.35(1.27-1.42) ***	0.89(0.72-1.09)
65~84	0.83(0.64-1.07)	1.56(1.44-1.70) ***	0.57(0.43-0.75) ***
85~	-	1.32(0.78-2.23)	-
Ethnic			
Han	1.19(1.07-1.31) **	1.16(1.12-1.20) ***	1.14(1.00-1.29)
Minority	1.15(0.98-1.34)	1.53(1.43-1.63) ***	1.03(0.86-1.24)
Unknown	-	0.45(0.11-1.95)	-
Occupation			
Preschooler	1.14(0.58-2.22)	1.39(0.57-3.39)	0.51(0.20-1.35)
Student	1.34(1.17-1.55) ***	0.93(0.83-1.05)	1.39(1.11-1.73) **

© 2024 Li T et al. *JAMA Network Open*.

Industrial worker	1.12(0.66-1.89)	1.16(0.97-1.40)	0.98(0.55-1.74)
Agricultural worker	1.34(1.17-1.52) ***	1.31(1.26-1.36) ***	1.11(0.96-1.28)
Commercial worker	1.19(0.45-3.17)	0.98(0.72-1.33)	1.39(0.46-4.26)
Healthcare worker	0.77(0.19-3.08)	0.99(0.44-2.21)	1.30(0.16-10.46)
Retired	0.51(0.33-0.79) **	1.23(0.98-1.55)	0.41(0.24-0.70) **
Office worker	1.76(0.66-4.70)	1.05(0.77-1.44)	2.67(0.95-7.53)
Domestic duties or unemployed	1.11(0.72-1.70)	1.47(1.28-1.69) ***	0.74(0.47-1.15)
Others	1.25(0.81-1.91)	1.22(1.03-1.46) *	1.05(0.63-1.73)
Migrant			
Yes	1.20(0.84-1.71)	1.12(1.01-1.24) *	1.31(0.88-1.95)
No	1.23(1.13-1.35) ***	1.29(1.25-1.34) ***	1.11(0.99-1.23)
Bacteriologically Result			
Positive	1.03(0.87-1.23)	1.21(1.15-1.28) ***	0.91(0.75-1.10)
Negative	1.27(1.15-1.41) ***	1.33(1.27-1.38) ***	1.21(1.06-1.38) **
Unknown	1.39(1.04-1.87) *	1.05(0.77-1.45)	1.32(0.81-2.16)
Rifampin Resistance			
Resistance	0.27(0.03-2.04)	0.76(0.18-3.13)	-
Sensitive	0.59(0.35-0.98) *	1.48(1.24-1.76) ***	0.49(0.27-0.88) **
Unknown	1.26(1.15-1.37) ***	1.26(1.22-1.30) ***	1.13(1.02-1.26) *
Therapeutic Regimen			
2HRZE/4HR	1.21(1.10-1.33) ***	1.26(1.22-1.31) ***	1.12(1.00-1.25) *
2HRZE/7-10HRE	1.08(0.50-2.31)	1.23(0.85-1.77)	0.78(0.34-1.79)
2HRZE/10HRE	1.06(0.26-4.30)	1.22(0.91-1.65)	0.95(0.23-3.92)

© 2024 Li T et al. *JAMA Network Open*.

Personalized	1.29(1.03-1.62) *	1.27(1.11-1.45) ***	1.06(0.72-1.56)
MDR	-	1.29(0.32-5.21)	-
Treatment management institutions			
Infectious disease hospital	1.52(0.85-2.71)	1.51(1.27-1.79) ***	1.07(0.53-2.16)
Primary health institutions	1.45(0.54-3.90)	1.04(0.65-1.66)	1.27(0.34-4.74)
Disease control institutions	1.33(1.20-1.46) ***	1.22(1.17-1.26) ***	1.25(1.11-1.41) ***
General hospitals	0.87(0.72-1.04)	1.46(1.34-1.58) ***	0.70(0.55-0.88) **
Supervision patterns			
Full-course supervision	1.26(1.07-1.47) **	1.37(1.32-1.43) ***	0.96(0.81-1.14)
Supervision in intensive phase	1.34(1.13-1.58) **	1.06(0.99-1.14)	1.39(1.13-1.70) **
Full-course management	1.15(0.99-1.33)	1.13(1.04-1.24) **	1.13(0.92-1.38)
Self-medication	1.08(0.80-1.48)	1.36(1.08-1.72) *	0.89(0.55-1.44)
Case finding pattern			
Direct visit to TB designated facilities	1.27(1.13-1.44) ***	1.18(1.12-1.24) ***	1.16(0.99-1.36)
Referral by non-TB designated facilities	1.24(1.07-1.45) **	1.32(1.25-1.38) ***	1.20(0.99-1.44)
Active screening	1.89(1.21-2.96) **	1.37(1.04-1.81) *	1.87(0.86-4.08)
Tracing by NTP	1.02(0.84-1.25)	1.39(1.27-1.53) ***	0.78(0.61-1.00)
2nd Month Sputum smear			
Positive	0.39(0.22-0.71) **	1.03(0.81-1.30)	0.42(0.19-0.90) *
Negative	1.26(1.14-1.39) ***	1.27(1.23-1.31) ***	1.13(1.01-1.27) *
Unknown	1.28(1.06-1.54) **	1.43(1.22-1.67) ***	1.11(0.82-1.50)
5th Month Sputum smear			
Positive	-	0.56(0.29-1.09)	-

© 2024 Li T et al. *JAMA Network Open*.

Negative	1.24(1.12-1.37) ***	1.28(1.24-1.32) ***	1.10(0.97-1.24)
Unknown	1.28(1.10-1.50) **	1.22(1.10-1.35) ***	1.17(0.93-1.46)
Patient Delay			
≤1 week	1.16(0.97-1.37)	1.22(1.13-1.32) ***	1.02(0.82-1.28)
1~4 weeks	1.34(1.13-1.61) **	1.29(1.21-1.38) ***	1.10(0.88-1.36)
＞4 weeks	1.20(1.07-1.35) **	1.28(1.23-1.33) ***	1.14(0.99-1.32)
Diagnostic Delay			
≤1 week	1.25(1.14-1.38) ***	1.26(1.22-1.31) ***	1.08(0.96-1.22)
＞1 week	1.12(0.95-1.32)	1.29(1.21-1.37) ***	1.21(0.97-1.50)
HIV Infection			
Positive	0.77(0.18-3.27)	1.92(1.38-2.67) ***	0.43(0.10-1.78)
Negative or unknown	1.22(1.12-1.32) ***	1.26(1.22-1.31) ***	1.11(1.00-1.23)
Treatment Outcome			
Cured	1.08(0.90-1.31)	1.20(1.14-1.27) ***	0.96(0.78-1.18)
Treatment completed	1.26(1.14-1.38) ***	1.32 (1.27-1.38) ***	1.18(1.04-1.33) *

*** suggested p<0.001; ** suggested p<0.01; * suggested p<0.05. PTB: pulmonary TB; - incapable coefficient

© 2024 Li T et al. *JAMA Network Open*.

eTable 4. TB Classifications: First Episode vs Recurrent Episode

	First episode		
	Primary PTB	Hematogenous Disseminated PTB	Secondary PTB
Total	584(100.0)	3834(100.0)	409518(100.0)
Recurrent episode			
Primary TB	74(12.7)	26(0.7)	256(0.0)
Hematogenous Disseminated TB	23(3.9)	299(7.8)	1903(0.5)
Secondary PTB	460(78.8)	3377(88.1)	399698(97.6)
Extrapulmonary TB	27(4.6)	132(3.4)	7661(1.9)

© 2024 Li T et al. *JAMA Network Open*.

eTable 5. Risk of Disease Classification, Drug Resistance, and Therapy Regimens

Therapeutic Regimen	Resistance	Sensitive	Unknown
Primary PTB			
2HRZE/4HR	1	1	1
2HRZE/7-10HRE	-	-	0.75(0.34-1.63)
2HRZE/10HRE	-	-	0.98(0.24-3.98)
personalized	-	-	0.78(0.60-1.02)
MDR	-	-	-
Hematogenous Disseminated PTB			
2HRZE/4HR	1	1	1
2HRZE/7-10HRE	-	0.88(0.22-3.62)	0.87(0.59-1.27)
2HRZE/10HRE	-	1.11(0.61-2.01)	0.83(0.59-1.19)
personalized	-	0.77(0.45-1.31)	0.83(0.72-0.97)*
MDR	-	0.82(0.09-7.40)	0.72(0.10-5.13)
Secondary PTB			
2HRZE/4HR	1	1	1
2HRZE/7-10HRE	0.44(0.14-1.39)	0.87(0.78-0.97)*	0.97(0.92-1.02)
2HRZE/10HRE	0.63(0.29-1.33)	0.75(0.70-0.81)***	0.86(0.82-0.90)***
personalized	0.53(0.36-0.79)**	0.83(0.80-0.87)***	0.93(0.92-0.94)***
MDR	0.27(0.08-0.91)*	0.74(0.65-0.85)***	0.92(0.80-1.05)

© 2024 Li T et al. *JAMA Network Open.*

eFigure 1. Group Process of Study

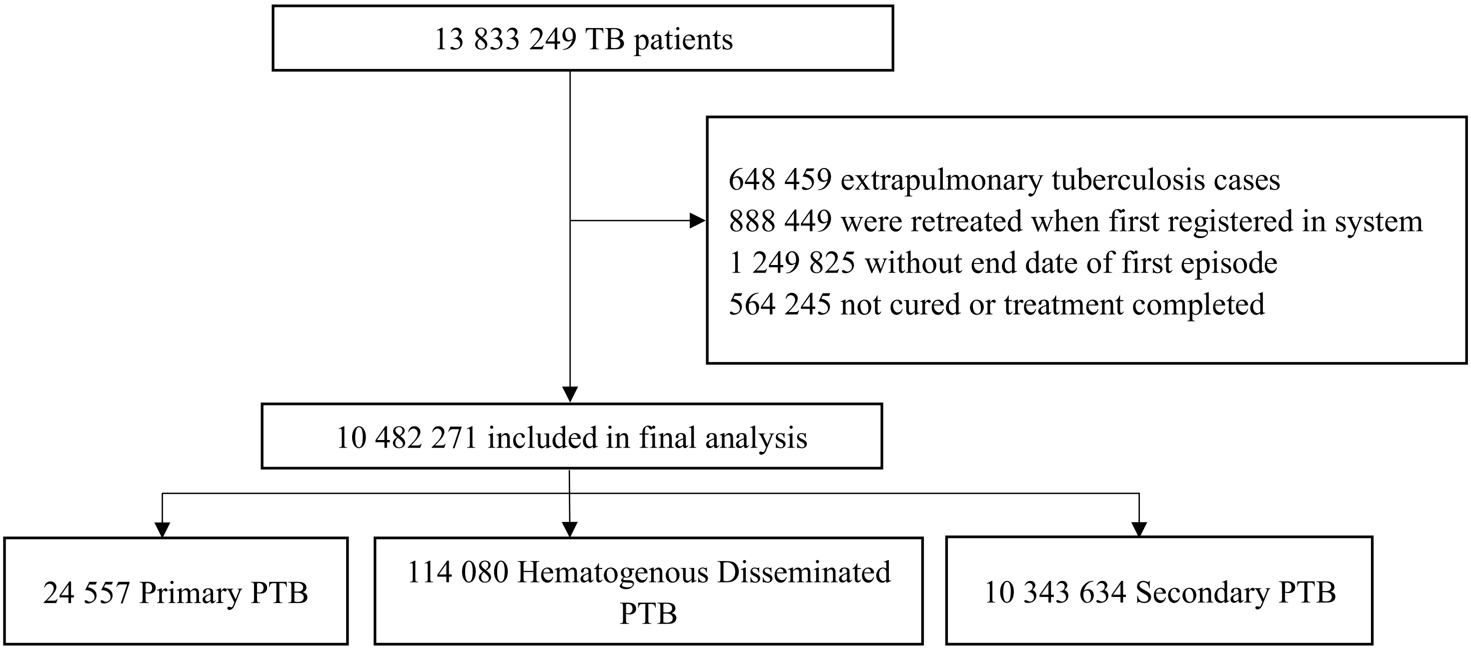

© 2024 Li T et al. *JAMA Network Open*.

eFigure 2. Cumulative Hazard of Recurrent PTB in China, Disaggregated by Subgroups, 2005 to 2021

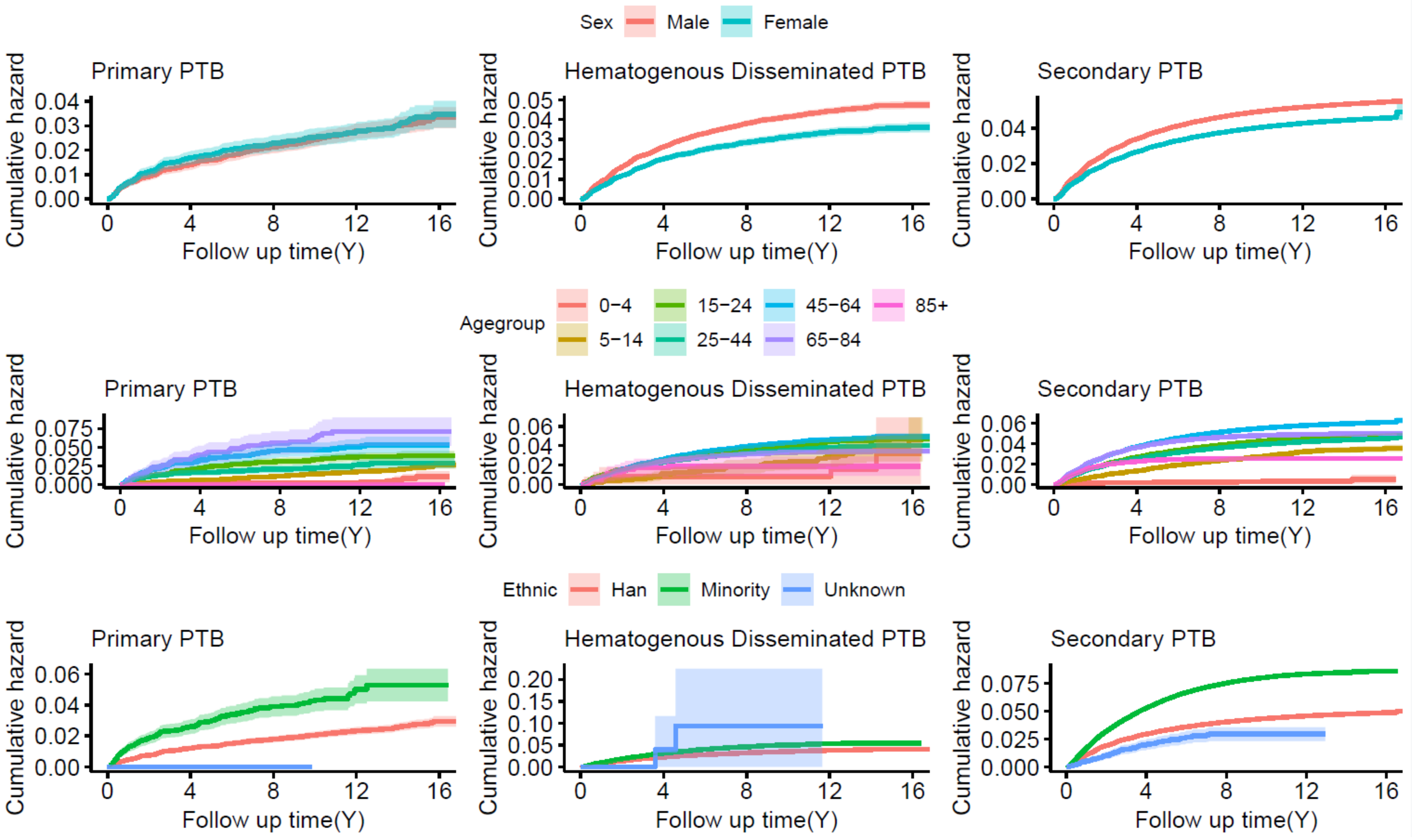

© 2024 Li T et al. *JAMA Network Open*.

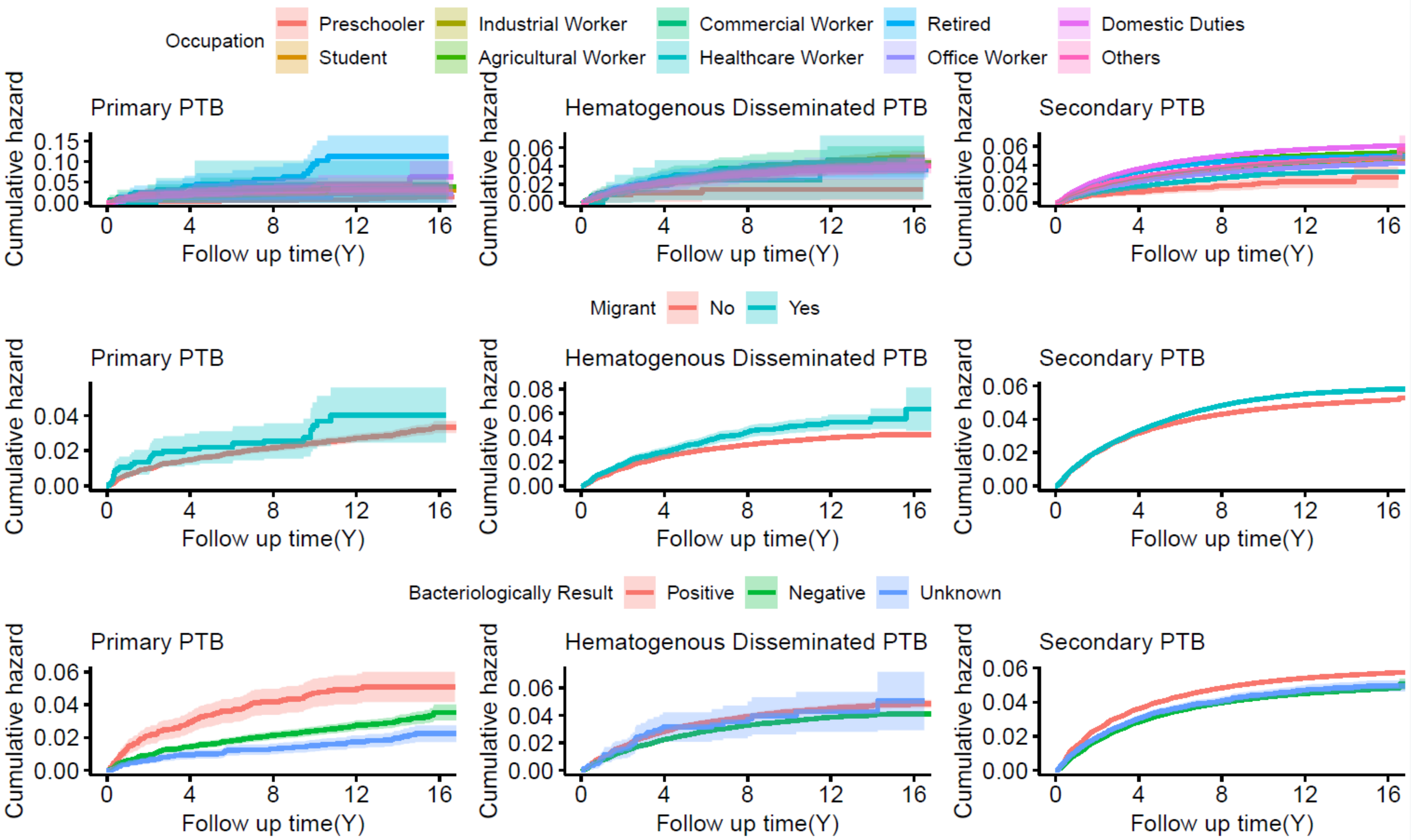

© 2024 Li T et al. *JAMA Network Open*.

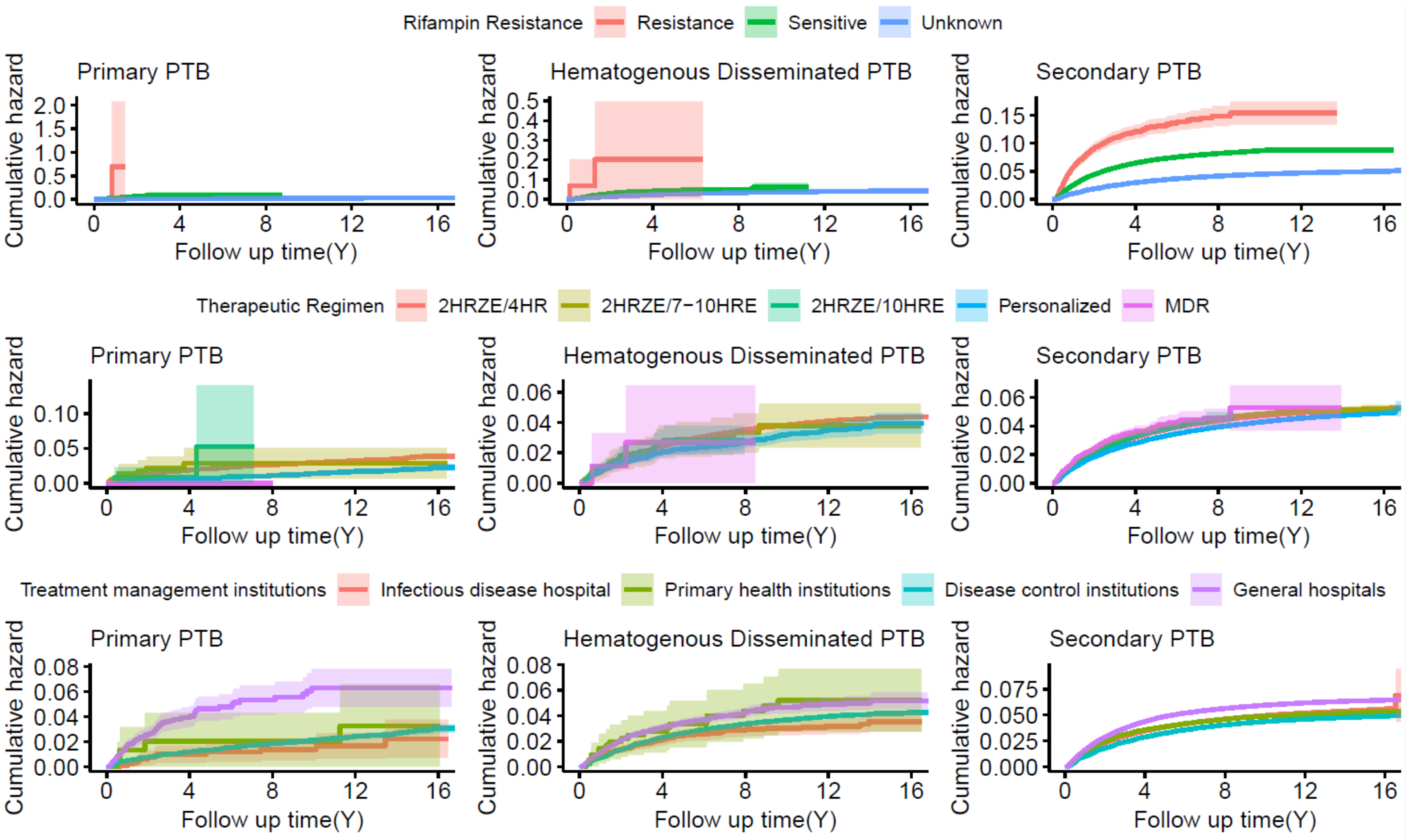

© 2024 Li T et al. *JAMA Network Open*.

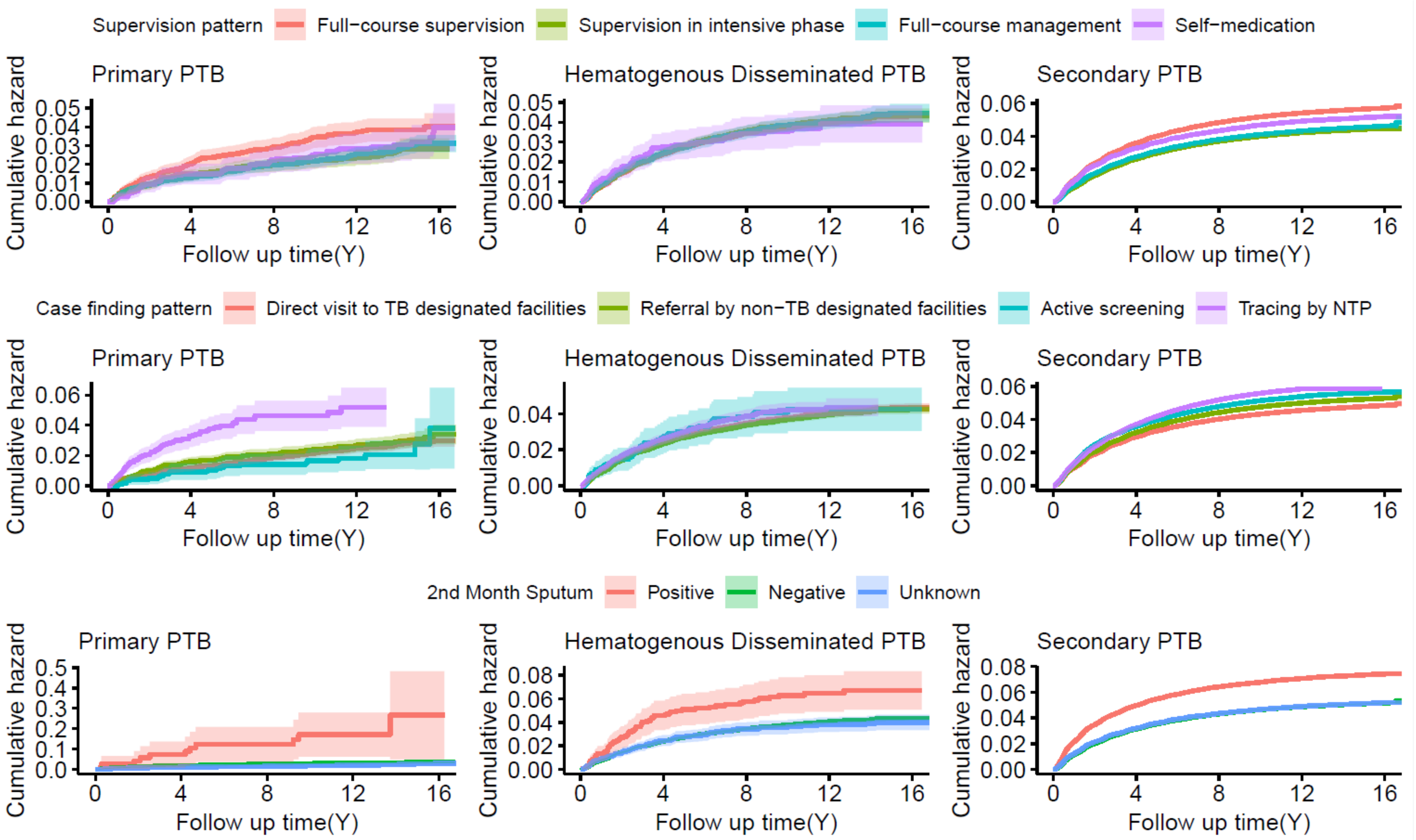

© 2024 Li T et al. *JAMA Network Open*.

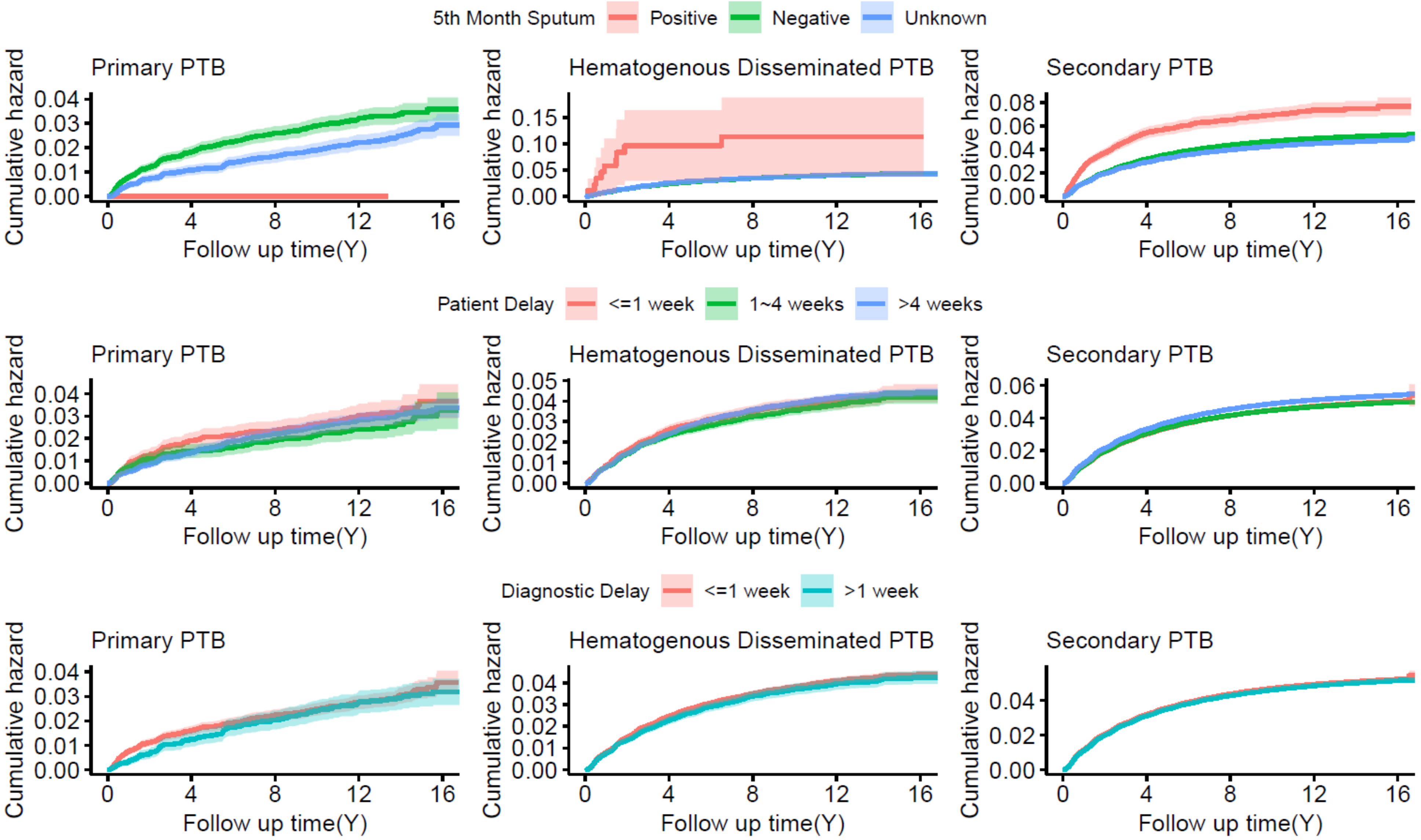

© 2024 Li T et al. *JAMA Network Open*.

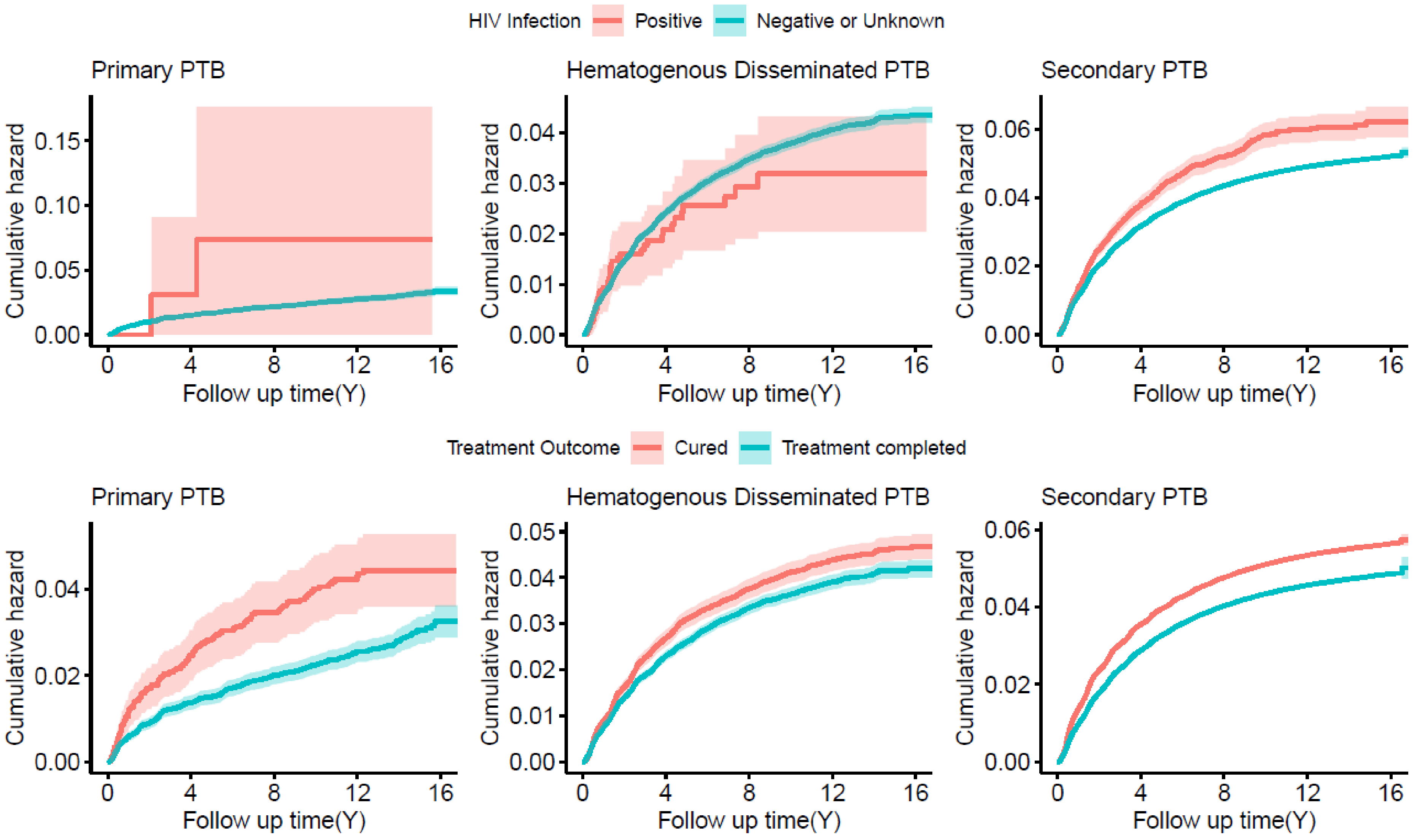

© 2024 Li T et al. *JAMA Network Open*.

eFigure 3. Recurrence Rate of i Year

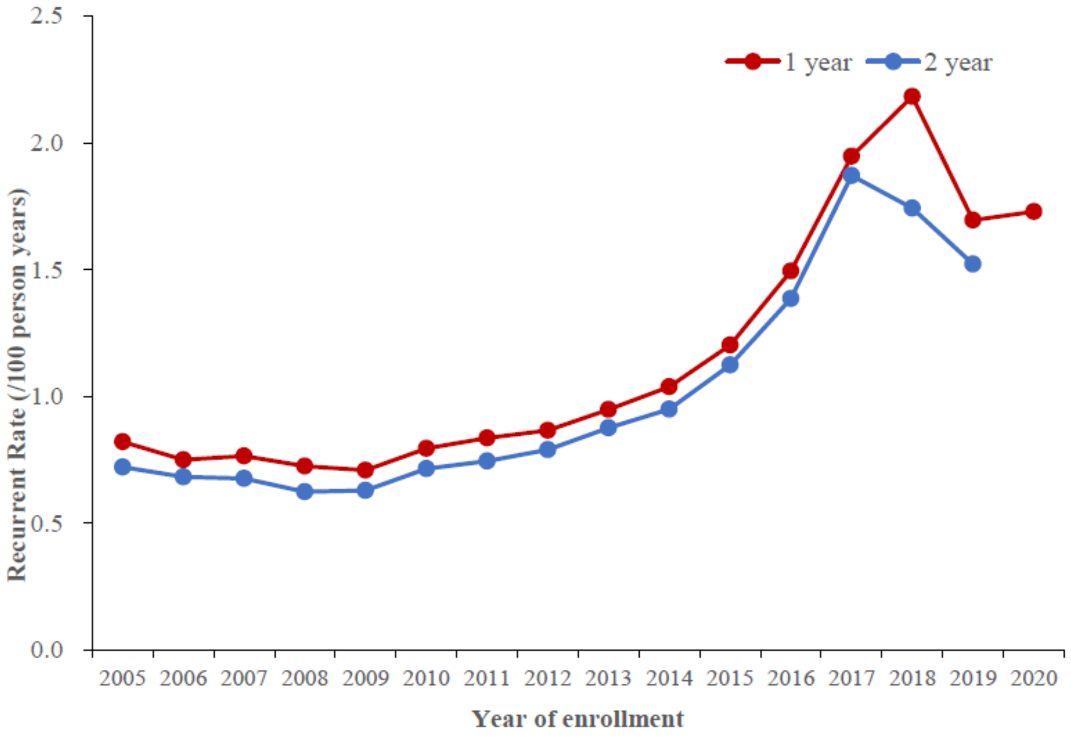

Figure S3. Recurrence rate of i year= The number of recurrent patients observed duration of n years /observed person years in i year   i: 2005~2020; n:1,2

© 2024 Li T et al. *JAMA Network Open*.

eFigure 4. Recurrence Rates in Different Provinces of China, 2005 to 2021

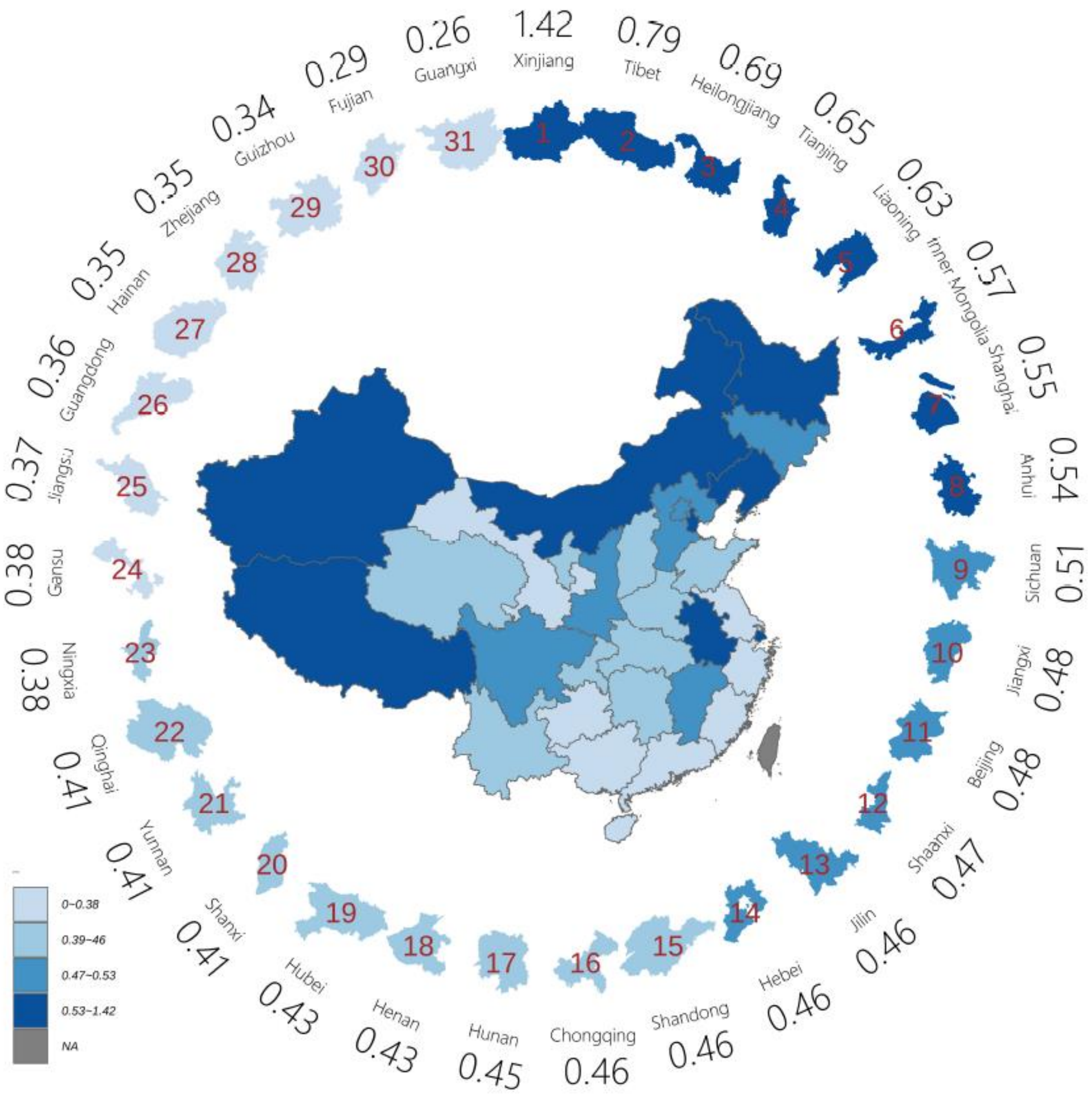

© 2024 Li T et al. *JAMA Network Open*.

eFigure 5. Schoenfeld Tests for Cox Proportional Risk Assumption

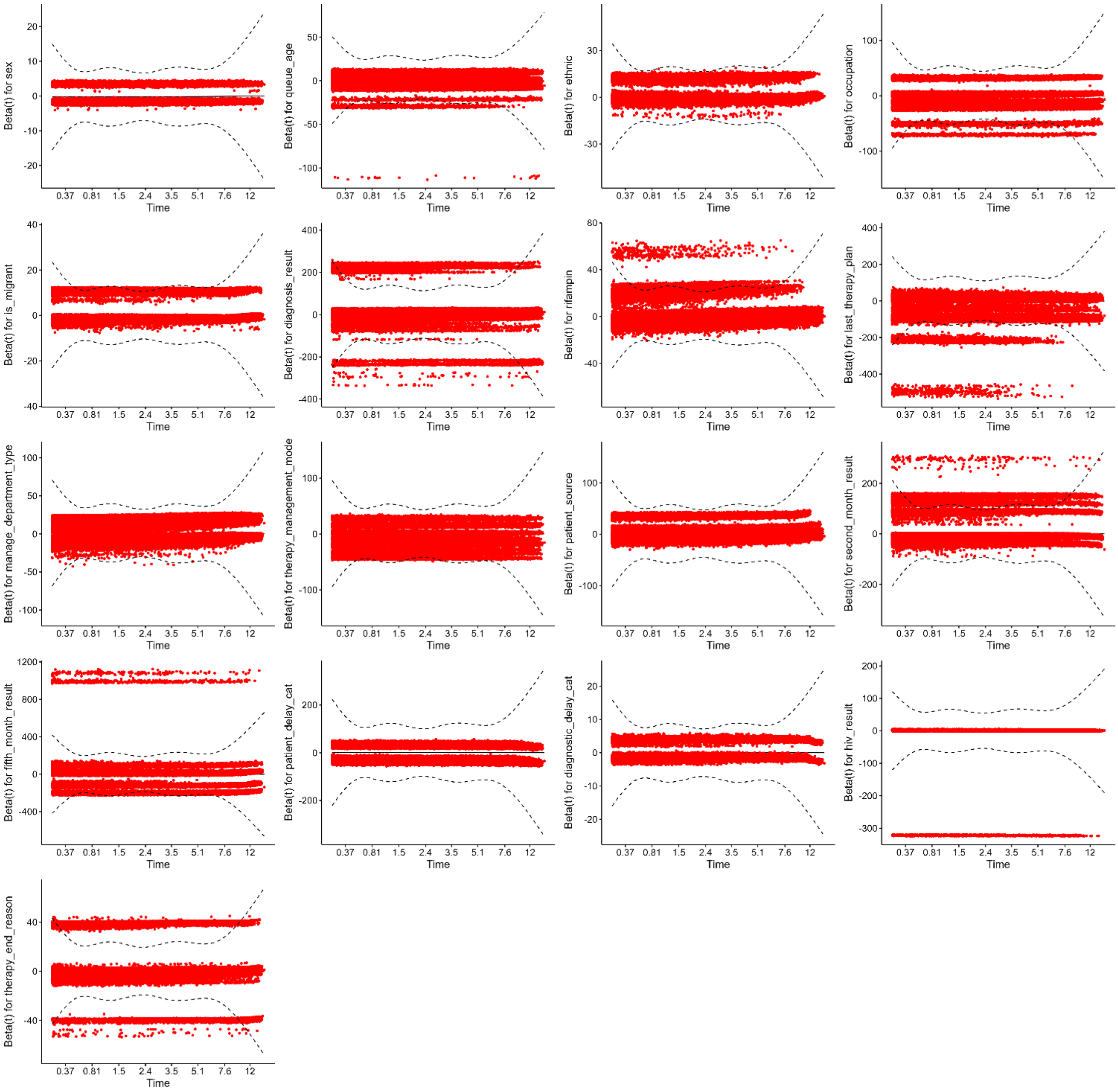

© 2024 Li T et al. *JAMA Network Open*.
