# Supplementary material for: Recurrent Pulmonary Tuberculosis in China, 2005 to 2021
Source: JAMA Netw Open. 2024 Aug 12;7(8):e2427266. doi: 10.1001/jamanetworkopen.2024.27266 (PMC11320166; doi:10.1001/jamanetworkopen.2024.27266)
Supplement: Supplement 2. — Data Sharing Statement [file jamanetwopen-e2427266-s002.pdf]

Data Sharing Statement

Li. Recurrent Pulmonary Tuberculosis in China, 2005 to 2021. *JAMA Netw Open*. Published August 12, 2024. doi:10.1001/jamanetworkopen.2024.27266

Data
Data available: No

Additional Information
Explanation for why data not available: The datasets used and/or analyzed during the present study are available from the corresponding author upon reasonable request.
